# Beneficial Effects of Long-Lasting Bicarbonate–Sulfate–Calcium–Magnesium Water Intake on Metabolic Dysfunction-Associated Steatotic Liver Disease (MASLD)-Related Outcomes via Impacting Intestinal Permeability (IP), IP-Related Systemic Inflammation, and Oxidative Stress

**DOI:** 10.3390/nu17213452

**Published:** 2025-10-31

**Authors:** Marcello Dallio, Mario Romeo, Fiammetta Di Nardo, Giusy Senese, Alessia Silvestrin, Annachiara Coppola, Carmine Napolitano, Paolo Vaia, Claudio Basile, Giuseppina Martinelli, Alessia De Gregorio, Alessandro Federico

**Affiliations:** Hepatogastroenterology Division, Department of Precision Medicine, University of Campania Luigi Vanvitelli, 80138 Naples, Italy; marcello.dallio@unicampania.it (M.D.); fiammetta.dinardo@studenti.unicampania.it (F.D.N.); giusy.senese@unicampania.it (G.S.); alessia.silvestrin@unicampania.it (A.S.); annachiara.coppola@studenti.unicampania.it (A.C.); carmine.napolitano1@studenti.unicampania.it (C.N.); paolo.vaia@studenti.unicampania.it (P.V.); claudio.basile@unicampania.it (C.B.); giuseppina.martinelli@studenti.unicampania.it (G.M.); alessia.degregorio2@studenti.unicampania.it (A.D.G.); alessandro.federico@unicampania.it (A.F.)

**Keywords:** hepatic steatosis, nutrients, intestinal permeability, inflammation

## Abstract

**Background/Objectives**: Fonte Essenziale^®^, a mineral water rich in bicarbonate, sulfate, calcium, and magnesium, has shown potential in modulating the gut–liver axis and microbiota in hepatic steatosis. However, its long-term effects on intestinal permeability (IP), systemic inflammation (SI), and oxidative stress—key factors in Metabolic dysfunction-associated steatotic liver disease (MASLD)—remain unexplored. **Methods**: Eighty-seven MASLD patients were randomized into two groups: group A received Fonte Essenziale^®^ (400 mL/day, fasting) plus a controlled nutritional regimen for 12 months, followed by a 6-month water washout; group B followed only the controlled nutritional regimen. IP markers, SI (IL-1β, IL-6, TNF-α), oxidative stress (dROMs/BAP), and clinical data (including Controlled Attenuation Parameter—CAP) were assessed at baseline (T0), 12 months (T12), and post-washout (T18). Baseline increased IP (in-IP) was defined by fecal zonulin > 110 ng/mL and serum LBPp > 10 µg/mL; improvement (im-IP) required normalization of both. A ≥30% CAP reduction indicated steatosis improvement. **Results**: Thirty-eight patients in group A and thirty-nine in group B completed the study. At T12, group A showed significant reductions in fecal zonulin (*p*: 0.0163) and serum LBPp (*p* < 0.0001), with increased occludin and claudin 1 (all *p* < 0.0001). Im-IP prevalence was higher in group A (*p*: 0.0037). Group A also showed significant reductions in IL-1β, TNF-α, IL-6, LPS, and dROM/BAP (all *p* < 0.05), especially among those with im-IP. CAP, insulin, and HDL levels improved significantly (all *p* < 0.0001). Multivariate analysis confirmed water intake (aOR: 2.185, *p*: 0.001) and im-IP achievement (aHR: 1.267, *p*: 0.021) as predictors of steatosis improvement. Benefits persisted at T18. **Conclusions**: Prolonged Fonte Essenziale^®^ intake improved hepatic steatosis and MASLD outcomes by modulating IP, SI, and oxidative stress. This trial has been registered on clinicaltrials.gov (NCT07211113).

## 1. Introduction

Metabolic dysfunction-associated steatotic liver disease (MASLD) has recently emerged as the most prevalent cause of chronic liver disease globally, reflecting a paradigm shift in the understanding of hepatic steatosis as a multisystemic metabolic disorder. MASLD is characterized by a complex interplay of hepatic and extrahepatic manifestations, prominently including insulin resistance (IR)-driven cardiometabolic comorbidities such as type 2 diabetes mellitus and dyslipidemia, [1], confirming its systemic nature [2,3,4].

Among the different pathogenetic drivers, increased intestinal permeability (IP) has been identified as a central determinant of the gut–liver axis [5,6]. Disruption of epithelial barrier integrity facilitates the translocation of pathogen-associated molecular patterns (PAMPs), such as lipopolysaccharide (LPS), into the portal circulation, leading to activation of hepatic innate immune cells and the perpetuation of low-grade chronic systemic inflammation (SI) and oxidative stress [7]. This inflammatory milieu further worsens IR, accelerating disease progression and ultimately contributing to the MASLD cardiometabolic burden [8,9,10]. Tight-junction proteins, including occludin and claudins, as well as regulators such as zonulin, play a pivotal role in maintaining intestinal epithelial integrity. Their dysregulation correlates with the severity of MASLD and its metabolic complications [10,11,12]. Zonulin, in particular, has been proposed as a biomarker of altered IP in dysmetabolic settings, showing relevant correlations with steatosis worsening [6,7,13].

Recent advances highlight the contribution of gut dysbiosis and barrier dysfunction to MASLD pathophysiology, suggesting that interventions capable of restoring IP may mitigate SI, oxidative stress, and metabolic impairment [8,14,15]. In this context, non-pharmacological approaches to modulating the gut–liver axis are increasingly attractive, especially given the lack of approved pharmacotherapies for early-stage MASLD [16,17]. In this sense, diet-based intervention has been shown to potentially modulate disease activity, largely via influencing the gut microbiota—a key intermediary between diet and immune function—by promoting eubiosis and enhancing intestinal barrier integrity [18].

Fonte Essenziale^®^, a bicarbonate–sulfate–calcium–magnesium, low-sodium mineral water sourced from Terme di Boario, has demonstrated promising metabolic effects in patients with hepatic steatosis. A previous 6-month intervention study reported beneficial modulation of gut microbial taxa and key metabolic mediators, including gastrointestinal hormones (Glucagon-like peptide-1-GLP-1, and Peptide Tyrosine–Tyrosine-PYY, among others) and hepatokines (e.g., fetuin-A, selenoprotein P), suggesting a potential role in improving metabolic homeostasis. These findings align with emerging evidence on the influence of mineral-rich waters on gut microbiota composition, bile acid metabolism, and enterohepatic signaling pathways [11]. Importantly, bicarbonate and sulfate-rich mineral waters have been shown to modulate luminal and intracellular pH, stimulate bile flow, and enhance intestinal motility, ultimately contributing to epithelial homeostasis [19,20]. In MASLD-murine models, calcium–sulfate–bicarbonate water intake has been associated with improved histopathological features and increased occludin expression in intestinal mucosa, suggesting a direct effect on tight junction stabilization [21]. These mechanisms are consistent with the role of mineral ions in regulating tight junction dynamics and epithelial permeability [22]. Despite the established link between gut microbiota and intestinal barrier integrity, the specific impact of Fonte Essenziale^®^ on IP, inflammation, and oxidative stress remains unexplored. Notably, the regression of metabolic benefits following a 6-month washout period appears consistent with an effect mediated by barrier modulation and highlights the need for longer-term investigations [11].

Considering this background, the present study evaluated the impact of 12-month daily Fonte Essenziale^®^ water intake, in addition to specialist-prescribed lifestyle modifications (both dietary and physical exercise), on IP, IP-related systemic inflammation, and oxidative stress in a cohort of MASLD patients, simultaneously exploring the potential relative effects on relevant clinical metabolic outcomes.

## 2. Materials and Methods

### 2.1. Experimental Design

This was a prospective, randomized, controlled clinical trial (NCT07211113) carried out at the Hepatogastroenterology Division of the University of Campania “Luigi Vanvitelli” (Naples, Italy). The study was conceived and conducted within the framework of the Ferrarelle–Vanvitelli research agreement (n. 390-01583577-30059—date 2 September 2023) [11], which previously allowed the exploration of mineral water as a non-pharmacological intervention in chronic liver disease.

Patients presenting with MASLD diagnostic criteria [17] were consequently considered as potentially eligible individuals. All patients who were potentially considered eligible received a Transient Elastography (TE) evaluation with a determination of Liver Stiffness Measurement (LSM) to primarily exclude patients presenting with LSM-defined advanced fibrosis (AF) (≥F3). Subsequently, eligible participants were randomly assigned in a 1:1 ratio into two study arms: Group A and Group B. Group A patients received 400 mL/day of Fonte Essenziale^®^ mineral water, taken every morning on an empty stomach for 12 consecutive months, in addition to a specialist-prescribed controlled nutritional regimen (see next dedicated subparagraph) followed by a 6-month water wash-out period of exclusively the controlled nutritional regimen. Group B patients followed the same 12-month specialist-prescribed controlled nutritional regimen without water supplementation.

The water dose (400 mL/day) and the fasting administration in the morning were chosen in accordance with the original protocol and prior trials [1], as gastric emptying is faster and absorption more consistent when the stomach is empty, potentially enhancing the interaction with the intestinal mucosa.

Three study time points were identified: baseline (T0), after 12 months (T12), and after wash-out (T18) (this last was exclusively for Group A patients)

At the three time-study points, anthropometric, clinical, and demographic evaluation (including age, sex, smoking habits, MASLD-related comorbidities, Body Mass Index—BMI—calculation), as well as routine biochemical variables and TE-assessed liver disease progression status data, including both LSM-defined fibrosis severity and Controlled Attenuation Parameter (CAP)-defined steatosis severity, were collected. At T12, a reduction of at least 30% in baseline CAP individual values defined an improvement of steatosis.

Moreover, at the three study time points, after collecting a 20 mL serum and 200 mg fecal sample, an IP assessment was performed and included the dosage of a panel of biomarkers: fecal zonulin (ng/mL); serum LPS binding-protein (LBPp) (µg/mL), reflecting systemic exposure to bacterial endotoxins; and serum occludin and claudin-1 (ng/mL), representing epithelial tight-junction proteins [13]. At T0, the simultaneous evidence of fecal zonulin > 110 ng/mL and serum LBPp > 10 µg/mL was adopted to define a priori an altered, and thus, increased, IP (in-IP) [6,13]. At T12, an improvement in intestinal permeability (im-IP) was defined as a simultaneous reduction in fecal zonulin < 110 ng/mL and serum LBPp < 10 µg/mL. This dual definition, including fecal and serum markers, for both in-IP and im-IP, was adopted because single biomarkers alone may not reliably capture barrier integrity status and functioning [6,13]. Occludin and claudin-1 were included as supportive markers of tight-junction integrity; their serum concentrations were measured and descriptively reported, but no predefined threshold values were applied for categorical classification of IP status. Their expression profiles were used to corroborate the primary zonulin/LBPp-based definition of altered or improved permeability [6,13].

IP-related SI was evaluated at the three study time points through assessing circulating levels of LPS (ng/mL) and pro-inflammatory cytokines [interleukin (IL)-1β, IL-6, and Tumor Necrosis Factor alpha (TNF-α) pg/mL] [8,9]. Finally, at the three study time points, Biological Antioxidant Potential (BAP) and derivatives of Reactive Oxygen Metabolites (dROM) tests were adopted as well-validated complementary tests to assess IP-related systemic oxidative stress in chronic liver and systemic disorders [23,24,25,26].

Evaluating variations in IP status after the 12-month intervention in Group A compared with Group B represented the main basic scientific objectives of this study. To evaluate the changes in IP-related SI biomarkers and oxidative stress serum levels in Group A compared with Group B according to variations in IP status constituted the secondary basic scientific objectives of this study. To investigate variations in hepatic steatosis severity in the two study groups simultaneously with changes in relevant biochemical metabolic variables represented the secondary clinical translational objectives of this research. Finally, limited to Group A, to evaluate changes in IP-related biomarkers, SI, and oxidative stress variables, as well as in clinical metabolic features and hepatic steatosis severity at the end of the wash-out period, represented ancillary outcomes of this study.

**Figure 1:**
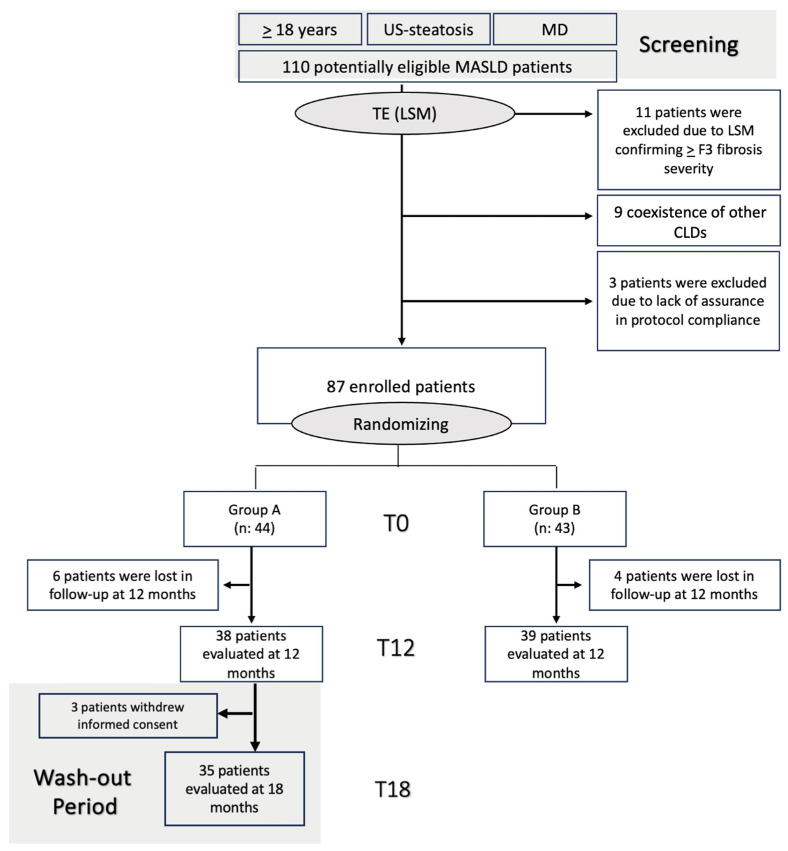
Flow chart summarizing the different phases of recruitment and follow-up. Patients presenting with age ≥ 18 years, ultrasound-based evidence of hepatic steatosis, and metabolic dysfunction (MD) were initially screened. MD was defined by at least 1 out of 5 of the following: (1) Body Mass Index ≥ 25 kg/m^2^; (2) fasting plasma glucose ≥ 100 mg/dL or HbA1c ≥ 5.7% or type 2 diabetes or treatment for type 2 diabetes; (3) blood pressure ≥ 130/85 mmHg or specific antihypertensive drug treatment; (4) plasma triglycerides ≥ 150 mg/dL or plasma High-density Lipoprotein (HDL)-cholesterol ≤ 40 mg/dL or lipid-lowering treatment. LSM was subsequently adopted to exclude patients with advanced fibrosis (≥F3). Selected patients were finally properly randomized into the two study groups. MASLD: Metabolic dysfunction-associated steatotic liver disease; MD: metabolic dysfunction; CLD: chronic liver disorders; US: ultrasound; TE: transient elastography; LSM: liver stiffness measurement.

The screening, enrollment, randomization, and follow-up is summarized in Figure 1. This trial has been registered on clinicaltrials.gov (NCT07211113; unique protocol 0001933/i/2023; accessed on 2 January 2023 https://register.clinicaltrials.gov/).

### 2.2. Participants

This study complies with the ethical guidelines of the Declaration of Helsinki (1975) and received approval from the ethical committee of the University of Campania Luigi Vanvitelli in Naples (protocol n. 0001933/i—date 20 January 2023). Patients were enrolled at the Hepato-Gastroenterology Division of the University of Campania Luigi Vanvitelli between February 2023 and September 2023.

Individuals aged ≥18 years with a diagnosis of MASLD were consecutively screened. According to recently proposed criteria, MASLD diagnosis was based on an imaging ultrasound of hepatic steatosis, with clinical and biochemical features confirming metabolic dysfunction (MD) [3] (Figure 1).

MD was defined by at least 1 out of 5 of the following: (1) body Mass Index ≥ 25 kg/m^2^; (2) fasting plasma glucose ≥ 100 mg/dL or HbA1c ≥ 5.7% or type 2 diabetes or treatment for type 2 diabetes; (3) blood pressure ≥ 130/85 mmHg or specific antihypertensive drug treatment; (4) plasma triglycerides ≥ 150 mg/dL or plasma High-density Lipoprotein (HDL)-cholesterol ≤ 40 mg/dL or lipid-lowering treatment. In detail, the inclusion criteria were as follows: age ≥ 18 years, imaging ultrasound-based evidence of hepatic steatosis, and clinical and biochemical features confirming MD (Figure 1) [3].

All patients who were potentially considered eligible received a TE evaluation with the LSM definition. Importantly, patients with AF (TE-assessed LSM confirming ≥F3) were excluded. This choice was motivated by evidence that in AF, IP is significantly altered, contributing to an enhanced bacterial translocation, both events influenced by the potential presence of portal hypertension [5,6].

Other exclusion criteria were as follows: (a) chronic liver disease (CLD) of other etiology [hepatitis B and/or C virus, alcohol-related liver disorder (ALD) with evidence of alcohol abuse history assessed by using the Alcohol Use Disorders Identification Test questionnaire [27], hemochromatosis, Wilson’s disease, or cholestatic liver disorders]; (b) evidence of cancer, including hepatocellular carcinoma (HCC); (c) acute systemic infections; (d) pregnancy; (e) psychological/psychiatric problems potentially invalidating the informed consent and/or conditions impacting the ability to adhere to dietary recommendations and intervention protocol; (f) chronic kidney disease (with Estimated Glomerular Filtration Rate—eGFR < 30 mL/min); (g) the use (ongoing and/or in the 3 months preceding the enrollment) of drugs/supplements that could influence IP (e.g., probiotics, antibiotics, NSAIDs).

#### 2.2.1. Randomization and Intervention

Eligible participants were randomly assigned in a 1:1 ratio into two study arms: (1) Group A: received 400 mL/day of Fonte Essenziale® mineral water, taken every morning on an empty stomach for 12 consecutive months, in addition to a specialist-prescribed controlled nutritional regimen followed by a 6-month water wash-out period of exclusively the controlled nutritional regimen; (2) Group B: followed the same specialist-prescribed controlled nutritional regimen without water supplementation.

The randomization list was generated using online randomization software (https://www.graphpad.com) (accessed on 2 January 2023) using permuted block list generation (to further reduce sequence predictability). Furthermore, the methods and tools used to generate the sequence were tracked. Regarding allocation concealment, the following criteria were also met: the block size was not disclosed to the investigators enrolling the patients; the investigators recruiting the patients did not know to which group the next patient was assigned. For this purpose, investigators not involved in patient enrollment prepared a numbered sequence of opaque, sealed envelopes containing the patient’s group (A or B) assignment. To prevent its subversion, the list remained inaccessible, and the envelopes were opened sequentially after patient enrollment and consent was obtained.

#### 2.2.2. Specialist-Prescribed Controlled Nutritional Regimen

All patients, regardless of group allocation, received a standardized hypocaloric Mediterranean-style diet designed to reduce total caloric intake by about 20–25% from daily requirements. Macronutrient distribution was approximately 45–50% carbohydrates, 25–30% fats (with <7% saturated fats), and 20–25% proteins. Fiber intake was encouraged, while refined sugars and ultra-processed foods were minimized [28]. Moreover, all patients were recommended to practice regular physical exercise, intended as at least >150 min/week of moderate physical activity, according to current clinical practice guidelines [17].

This nutritional approach was guided by two solid evidence-based reasons: on one side, a uniform background intervention appeared crucial to minimize lifestyle habits-dependent intrapopulation study variability that would have confounded gut dysbiosis/IP-related and inflammatory outcomes; on the other, Mediterranean-style diets and proper physical exercise have been consistently associated with improved metabolic control in MASLD patients, both representing a cornerstone in the management of these patients [17,28,29,30].

Dietary compliance was evaluated through weekly food diaries, which were reviewed by dedicated nutritionists. In particular, the Winfood Software 2.0 package (Medimatica s.r.l., Martinsicuro, Italy) software analyzed daily meal intake (by examining both food types and quantities) based on each patient’s entries in a dedicated electronic diary. By performing this analysis, the percentage of macronutrients and micronutrients present in each food, the daily intake measured in grams per kilogram of body weight (g/kg), and the total energy consumption (kcal/day) over an entire week, including weekends, factoring in both the quantity and quality of the consumed foods, were calculated. Adherence was classified as high (>80% adherence), moderate (50–79%), or low (<50%), based on the percentage of prescribed diet components met.

Finally, the validated “International Physical Activity Questionnaire Short Form” (IPAQ-SF) was adopted to assess the compliance with physical activity recommendations in terms of time spent on moderate physical exercise (File S1).

#### 2.2.3. General Compliance Assessment

Patients in both arms received monthly telephone calls to reinforce adherence and troubleshoot practical barriers. In addition, structured questionnaires were administered at the 6- and 12-month visits to formally assess the frequency, timing, and tolerability of water intake, ensuring consistency with protocol requirements (400 mL/day in the morning on an empty stomach). Where feasible, bottle counts were performed to provide an objective cross-check of self-reported intake.

The next dedicated subparagraphs report in detail the collected variables and the analyses performed at the different study points.

### 2.3. Assessment of Anthropometrical, Clinical, and Biochemical Variables

Anthropometrical parameters collected included the determination of BMI by dividing the patient’s weight by the square of their height (kg/m2), SBP (mmHg), and diastolic blood pressure (DBP) (mmHg). Clinical evaluation included the collection of complete medical history and the assessment of alcohol consumption, nutritional habits (including physical exercise and dietetic attitudes), smoking, drug abuse, and baseline and follow-up therapy records (antiaggregant/anticoagulant medications, statins, and other MASLD-related metabolic drugs, including glucagon-like peptide-1 receptor agonist GLP1-RA, and Sodium-Glucose Transport 2 inhibitors—SGLT2-i—administration) for each patient.

The collected biochemical data were as follows: aspartate aminotransferase (AST), alanine aminotransferase (ALT), gamma-glutamyl transferase (GGT), alkaline phosphatase (ALP), total bilirubin (TB), platelet count (PLT), plasma albumin (PA), prothrombin time (PT) total cholesterol (TC), HDL, low-density lipoprotein (LDL) cholesterol, TG, high-sensitivity C-reactive protein (CRP), insulin (μU/mL), FPG, and glycosylated hemoglobin (HbA1c) (%). The homeostatic model assessment for insulin resistance (HOMA-IR) was calculated by using the validated formula: fasting insulin (μU/mL) × FPG (mmol/L)/22.5 [31].

All biochemical parameters were measured using standard automated laboratory procedures. Liver enzymes (AST, ALT, GGT, ALP), TB, PA, TC, HDL, LDL, and TG were quantified via enzymatic colorimetric assays on a Roche Cobas® 8000 modular analyzer (Roche Diagnostics, Mannheim, Germany). PLT and PT were assessed using an automated hematology analyzer (Sysmex XN-Series, Sysmex Corporation, Kobe, Japan). CRP levels were determined by high-sensitivity immunoturbidimetric assay on the same Roche platform. FPG and insulin were measured using enzymatic and electrochemiluminescence immunoassay (ECLIA) (Roche Diagnostics, Basel, Switzerland) methods, respectively. HbA1c was quantified by high-performance liquid chromatography (HPLC) using the Bio-Rad D-10 system (Bio-Rad Laboratories, Hercules, CA, USA). All assays were performed in accordance with manufacturer protocols and internal quality control procedures, and results were interpreted using reference ranges validated by the institutional laboratory.

### 2.4. Abdominal Ultrasound and Transient Elastography

B-mode ultrasonography was conducted by an experienced physician to assess hepatic echogenicity and identify the presence of a bright liver. Evaluation was based on the liver-to-kidney echogenicity ratio, complemented by the visualization of hepatic veins and the diaphragm. Imaging was performed using the GE Logiq E10^®^ ultrasound system. Liver stiffness measurement (LSM) was subsequently carried out using FibroScan^®^ [version 502; Echosens, Paris, France], employing both M and XL probes according to patient characteristics [32]. The XL probe was selected when the ultrasound-determined distance between the skin and the liver capsule exceeded 2.5 cm and/or the patient’s body mass index (BMI) was greater than 30. LSM was performed by a trained physician, with successful acquisition defined as the attainment of 10 valid measurements. Measurement reliability was classified according to the criteria proposed by Boursier et al., as follows: “very reliable” (interquartile range to median ratio [IQR/M] ≤ 0.1), “reliable” (0.1 < IQR/M ≤ 0.3 or IQR/M > 0.3 with a median liver stiffness < 7.1 kilopascals [kPa]), and “poorly reliable” (IQR/M > 0.3 with a median liver stiffness ≥ 7.1 kPa) [32,33]. The following LSM cut-off scores were used to identify the different liver fibrosis stages according to the Metavir score: (a) F0–F2 ≤ 9.6 kPa; (b) F3: 9.7–13.5 kPa; (c) F4 ≥ 13.6 kPa [34]. The extent of hepatic steatosis was quantitatively assessed using the controlled attenuation parameter (CAP). CAP evaluates ultrasonic attenuation at a frequency of 3.5 MHz, utilizing signals acquired through the FibroScan^®^ M and XL probes, in accordance with established physical principles described elsewhere [33,35]. The CAP was measured only on validated measurements according to the same criteria used for LSM [32,33,35]. Based on CAP scores, patients were stratified according to the degree of hepatic steatosis as follows: S0, no steatosis (0–10% fat content; 0–237 dB/m); S1, mild steatosis (11–33% fat; 238–259 dB/m); S2, moderate steatosis (34–66% fat; 260–292 dB/m); and S3, severe steatosis (>67% fat; ≥293 dB/m). These thresholds were derived from the quantification of ultrasonic signal attenuation obtained from TE [32,33,35].

The operator was blinded to group allocation, and the same experienced physician performed all measurements to ensure consistency and minimize inter-operator variability.

### 2.5. Assessment of Intestinal Permeability Markers

Fecal zonulin concentrations were measured using a quantitative sandwich enzyme-linked immunosorbent assay (ELISA) (Zonulin ELISA Kit, Cat. No. K5600; Immundiagnostik AG, Bensheim, Germany) following the manufacturer’s instructions. Stool samples were collected in sterile polypropylene containers, immediately frozen at −80 °C, and thawed only once prior to analysis. Each sample was homogenized in extraction buffer, centrifuged at 10,000× *g* for 10 min at 4 °C, and the supernatant was used for the assay. The kit’s analytical sensitivity was 0.22 ng/mL, with intra-assay and inter-assay coefficients of variation (CVs) below 8% and 10%, respectively. Serum levels of claudin-1 and occludin were determined using human-specific ELISA kits (Claudin-1 ELISA Kit, Cat. No. E-EL-H5586; Occludin ELISA Kit, Cat. No. E-EL-H5587; Elabscience Biotechnology Inc., Houston, TX, USA). Venous blood samples were collected after overnight fasting, allowed to clot at room temperature, and centrifuged at 3000× *g* for 10 min. Serum aliquots were stored at −80 °C until analysis. Both kits had detection limits of 0.1 ng/mL, with intra-assay CVs < 6% and inter-assay CVs < 9%. LPS-binding protein (LBP) was quantified using the Human LBP ELISA Kit (Cat. No. HK315; Hycult Biotech, Uden, The Netherlands). Serum samples were processed identically to those used for tight junction protein assays. The kit’s sensitivity was 0.4 ng/mL, with intra-assay and inter-assay CVs of 5.5% and 7.8%, respectively. All assays were performed in duplicate, and absorbance was read at 450 nm using a calibrated microplate reader (BioTek Synergy HTX, Agilent Technologies, Santa Clara, CA, USA). Concentrations were calculated from standard curves generated using serial dilutions of known calibrators provided with each kit. Quality control samples were included in each run to ensure analytical consistency.

### 2.6. Assessment of Systemic Inflammation Markers

Serum concentrations of IL-1β, IL-6, and TNF-α were measured using high-sensitivity enzyme-linked immunosorbent assay (ELISA) kits specific for human cytokines (IL-1β: Cat. No. E-EL-H0149; IL-6: Cat. No. E-EL-H0102; TNF-α: Cat. No. E-EL-H0109; Elabscience Biotechnology Inc., Houston, TX, USA).

Peripheral venous blood samples were collected in serum separator tubes following overnight fasting, allowed to clot at room temperature for 30 min, and centrifuged at 3000× *g* for 10 min. Serum aliquots were stored at −80 °C and thawed only once prior to analysis. All assays were performed in duplicate according to the manufacturer’s protocols. Optical density was measured at 450 nm using a calibrated microplate reader (BioTek Synergy HTX; Agilent Technologies, Santa Clara, CA, USA), and cytokine concentrations were calculated from standard curves generated using serial dilutions of recombinant human standards provided with each kit. The analytical sensitivity was 1.56 pg/mL for IL-1β, 0.94 pg/mL for IL-6, and 4.69 pg/mL for TNF-α. Intra-assay coefficients of variation (CVs) were <6% for all cytokines, and inter-assay CVs were <9%. Quality control samples and blank wells were included in each run to ensure analytical precision and background correction. Reference ranges for healthy individuals, as reported by the manufacturer, were as follows: IL-1β, <5 pg/mL; IL-6, <7 pg/mL; TNF-α, <8 pg/mL. Values below the lower limit of quantification were excluded from analysis, and no samples exceeded the upper detection limit.

### 2.7. Assessment of Systemic Oxidative Stress and Antioxidant Capacity

The d-ROMs assay (colorimetric determination of Reactive Oxygen Metabolites^®^) and the BAP assay (Biological Antioxidant Potential^®^) were employed to evaluate systemic oxidative stress and antioxidant capacity, respectively, in accordance with the manufacturer’s protocol (Diacron International, Grosseto, Italy) [25]. Venous blood samples were collected, and serum was isolated by centrifugation at 2000 rcf for 10 min at room temperature.

The d-ROMs assay quantifies the intensity of the resulting chromogenic complex photometrically, which is directly proportional to the hydroperoxide concentration. One unit of measurement (U-CARR) corresponds to the quantity of hydroperoxides reducible by superoxide dismutase to approximately 0.08 mg/dL of H_2_O_2_. The established reference range is 20–24 mg/dL H_2_O_2_. Intra-assay and inter-assay coefficients of variation range from 0.3% to 6.6% and 0.3% to 5.1%, respectively.

Conversely, the BAP assay is based on the ability of the biological sample to reduce ferric ions (Fe^3+^) to their ferrous form (Fe^2+^). The reaction endpoint is measured photometrically at 505 nm. Results are expressed as µmol of antioxidant equivalents capable of reducing ferric ions per liter of sample, with normal values exceeding 2200 µmol/L. Both assays were conducted in duplicate using 96-well microplates (Greiner Bio-One, Kremsmünster, Austria) via an endpoint protocol, incorporating low- and high-serum controls and an internal calibrator supplied by Diacron International. Absorbance readings were obtained using a TECAN INFINITE M PLEX microplate reader (Tecan, Grödig, Austria).

The dROMs/BAP ratio was calculated considering the following interpretations: <0.1 (favorable redox balance), 0.1–0.2 (compensated oxidative stress), and >0.2 (severe oxidative stress).

### 2.8. Statistical Analysis

Continuous data were described as means and standard deviations (means ± SD), while categorical variables were described as n (%). The Kolmogorov–Smirnov test for normality evaluated whether parametric or non-parametric analysis should be applied. Between-group comparisons were made with Student’s *t*-test or the Mann–Whitney U test as appropriate; within-group comparisons were made with paired *t*-test or Wilcoxon signed-rank test. Categorical data were analyzed with χ^2^ or Fisher’s exact test. The McNemar test was adopted to assess significant differences in the proportion of patients obtaining im-IP in the two study groups at the two different study points. To identify independent predictors of hepatic steatosis improvement at T12 (binary outcome: improvement vs. no improvement), univariate and multivariate logistic regression analyses were performed, adjusting for sex, age, smoking, BMI, diabetes, MASLD-related drugs (statins, GLP1-RA, and SGLT2-i administration), and baseline CAP. Variables with *p* < 0.10 in univariate analysis were considered for inclusion in the multivariate model. Results are reported as odds ratios (OR) and adjusted odds ratios (aOR) with corresponding 95% confidence intervals (CI).

The sample size was estimated by using a chi-square test confronting two independent proportions, singularly predicting a 30% difference in the prevalence of subjects reaching an improvement in intestinal permeability (im-IP) in the water-receiving group compared to the control (significance: 0.05, type II error: 0.1; power: 0.9) (STATA14 for MacOS) and resulted in n. 38 individuals for each group.

Statistical significance was defined as *p* < 0.05 in a two-tailed test with a 95% confidence interval (C.I.). Statistical Program for Social Sciences (SPSS^®^) vs.18.0 was used to perform the analysis.

## 3. Results

### 3.1. Enrollment of MASLD Patients, Follow-Up, and Compliance Evaluation

A total of 110 MASLD patients were initially screened and considered for enrollment. However, 11 patients were excluded due to LSM confirming ≥F3 fibrosis severity, as well as 9 individuals due to the coexistence of other CLDs. Finally, 3 subjects were excluded due to their inability to guarantee adherence to the prescribed intake of Fonte Essenziale^®^ water; 87 MASLD patients were finally enrolled (Figure 1). The primary reason reported for the three excluded participants was the absence of a stable residence due to frequent work-related travel.

After randomization, 44 individuals were assigned to Group A and 43 subjects were assigned to Group B. Considering the 87 enrolled patients, after baseline (T0) evaluations, 38 individuals and 39 subjects belonging to Group A and Group B, respectively, accepted the invitation to return for a visit at T12, and 35 of the Group A individuals completed the wash-out period and were evaluated at T18 (3 patients withdrew informed consent and were lost to the follow-up at T18).

An overall low loss-of-compliance rate was reported (11.49%), and no statistically significant differences emerged in the specific comparison of the study groups (Group A: 13.63% vs. Group B: 9.31%; chi-square, *p*: 0.738). Participants adhered to the recommended dosage of the delivered water in 88.51% of instances and uniformly reported a positive sensory experience during consumption.

Figure 1 illustrates the flowchart detailing the sequential phases of patient enrollment, intervention, and follow-up (Figure 1).

### 3.2. Baseline Evaluations: Characteristics of the Study Groups

Of 87 enrolled patients, 50 (57.47%) were male, and the overall mean age was 53.49 years (mean ± SD: 53.49 ± 17.40). No statistically significant differences emerged in the comparison of sex distribution, age (mean ± SD), and proportion of smokers among the two study groups (Table 1). A similar prevalence of all MASLD-related cardiometabolic comorbidities (type 2 diabetes mellitus, obesity, essential arterial hypertension, and dyslipidemia) was reported in Group A compared with Group B (Table 1). Consistently, at baseline (T0), comparing the two study groups, no statistically significant differences were highlighted in BMI values, biochemical metabolic-related variables, and non-invasive tools assessing liver disease progression status (both LSM and CAP) (Table 1). Moreover, lifestyle habits were not different between the two study groups, revealing, also throughout the 12-month period, a similar time dedicated to daily physical exercise, as well as a comparable daily food and calorie intake (Table S1A).

Concerning the intestinal permeability markers, no differences emerged between the two groups except for higher levels of fecal zonulin highlighted in Group A (*p*: 0.04). A similar proportion of patients presenting with altered intestinal permeability (in-IP) emerged in Group A (19/44 individuals, 43.18%) compared with Group B (16/43, 37.20%) (chi-square, *p*: 0.663). Finally, focusing on SI and oxidative stress, no differences in LPS, IL-1β, IL-6, and TNF-α serum levels, or in dROMs/BAP ratio, emerged in the comparison of the two study groups.

Table 1 presents the complete demographic, anthropometric, clinical, biochemical, IP-related, SI-related, and oxidative stress-related data for the two study groups (Table 1).

**Figure 2:**
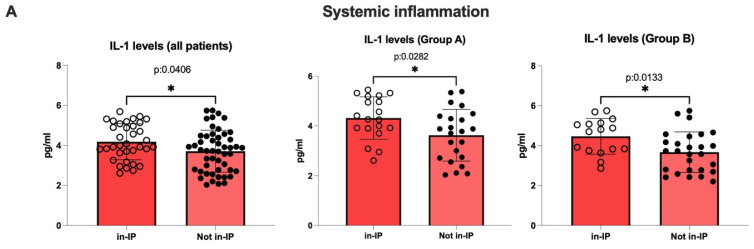

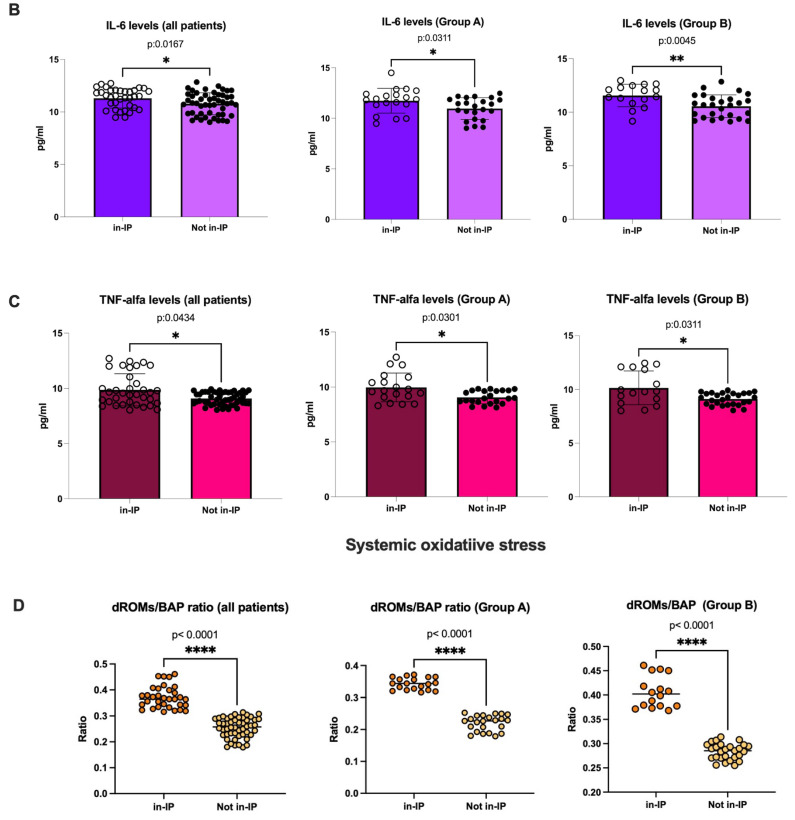

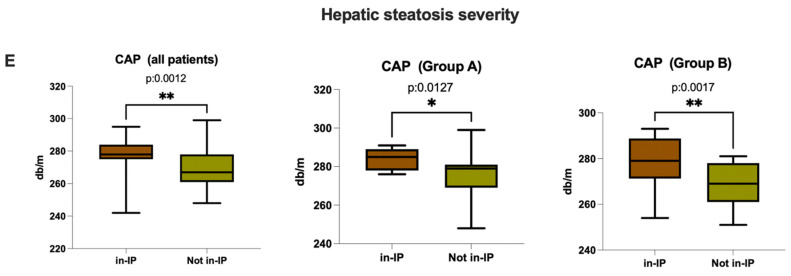
Systemic inflammation, oxidative stress, and hepatic steatosis severity according to the baseline intestinal permeability status. At baseline, an altered integrity of the bowel barrier was defined as increased intestinal permeability (in-IP); in contrast, a preserved integrity of the gut barrier was defined as not-increased intestinal permeability (not in-IP). (**A**) Serum levels of IL-1β according to intestinal permeability status in all patients, Group A, and Group B individuals; (**B**) Serum levels of IL-6 according to intestinal permeability status in all patients, Group A, and Group B individuals; (**C**) Serum levels of TNF-α according to intestinal permeability status in all patients, Group A, and Group B individuals; (**D**) dROMs/BAP ratios according to intestinal permeability status; (**E**) CAP values according to intestinal permeability status in all patients, Group A, and Group B individuals; */**/**** Mann–Whitney test analysis; IL: interleukin; CAP: controlled attenuation parameter; in-IP: increased intestinal permeability; Not in-IP: not-increased intestinal permeability; dROMs: derivatives of Reactive Oxygen; BAP: Biological Antioxidant Potential.

Finally, as expected, in line with the evidence supporting the role of altered intestinal permeability in contributing to chronic SI status, the serum levels of all pro-inflammatory cytokines and dROMs/BAP ratios were significantly higher in in-IP patients compared with individuals showing a preserved intestinal permeability status [all patients, IL-1β, *p*: 0.0406, IL-6, *p*: 0.0167, TNF-alfa, *p*: 0.0434; Group A, IL-1β, *p*: 0.0282, IL-6, *p*: 0.0311, TNF-alfa, *p*: 0.0301; Group B, IL-1β, *p*: 0.0133, IL-6, *p*: 0.0045, TNF-α, *p*: 0.0311; all patients, Group A, Group B, dROMs/BAP ratio, all *p* < 0.0001] (Figure 2A–D). Interestingly, hepatic steatosis (assessed by CAP) was also significantly more severe in patients showing altered intestinal permeability compared with subjects exhibiting a preserved bowel integrity [all patients, CAP, *p*: 0.0012; Group A, CAP, *p*: 0.0127; Group B, CAP, *p*: 0.0017] (Figure 2E).

### 3.3. 12-Month Follow-Up Evaluations

Table 2 reports all the 12-month follow-up evaluations relative to Group A and Group B, as well as the comparison between the assessed variables at the first two study time points (T0 and T12) (Table 2).

#### 3.3.1. Evaluation of Intestinal Permeability, Systemic Inflammation, and Oxidative Stress

**Figure 3:**
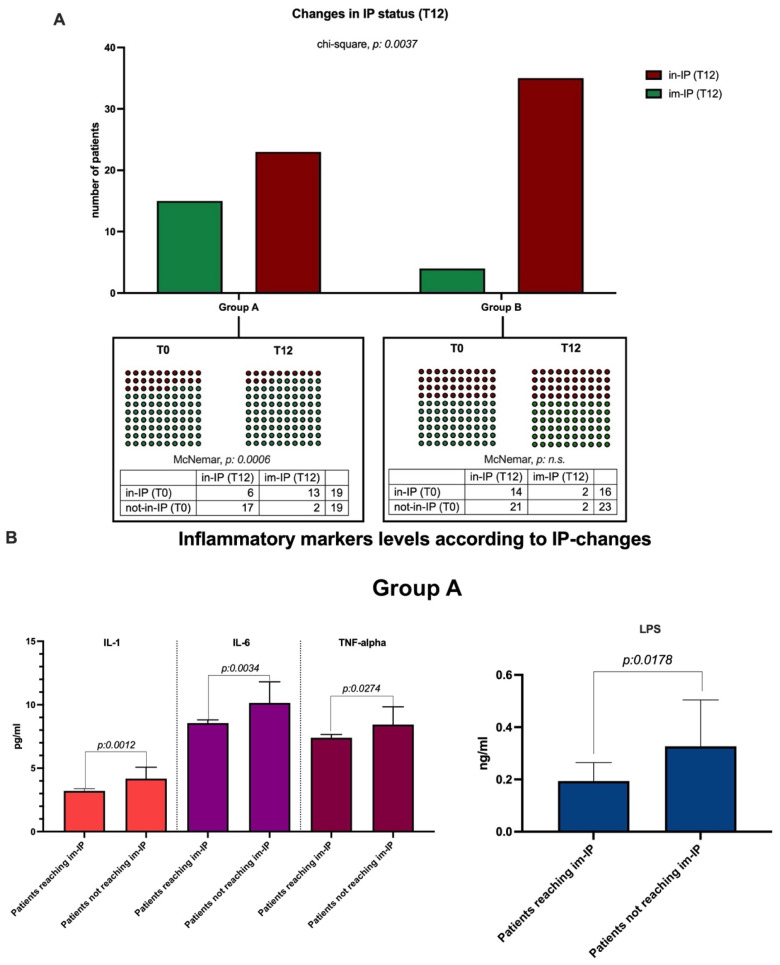
Changes in intestinal permeability (IP) status and IP-related systemic inflammation after the 12-month interventions. Two different conditions were identified: patients maintaining an altered intestinal permeability status (in-IP) and individuals gaining an improvement in the integrity of the intestinal barrier (im-IP) after the 12-month interventions. (**A**) The comparison of the proportion of patients reaching and not reaching an improvement in intestinal permeability in Group A vs. Group B (considering 6 patients and 4 patients were lost to follow-up, respectively, in Group A and Group B). In-iP: increased intestinal permeability; im-IP: improved intestinal permeability; (**B**) variations in serum levels of systemic inflammation markers (IL-1, IL-&, and TNF-alpha) in Group A according to the changes in intestinal permeability status. Mann–Whitney test analysis; IL: interleukin. TNF: Tumor Necrosis Factor; LPS: lipopolysaccharide; im-IP: improved intestinal permeability.

Unlike Group B, in Group A, at T12, a statistically significant reduction in fecal zonulin (*p*: 0.0163) and serum LPBp (*p* < 0.0001), simultaneously with an increase in serum occludin and claudin-1 levels (both, *p* < 0.0001) was observed. Consistently, in this group, the prevalence of patients (15/38, 39.47%) reaching im-IP (i.e., positively changing their intestinal permeability status) was significantly higher compared with group B (4/39, 10.25%) (chi-square, *p*: 0.0037) (McNemar, Group A T0 vs. T12, in-IP vs. im-IP, *p*: 0.0006) (Figure 3A).

Moreover, unlike Group B, IL-1β, TNF-α, IL-6, and LPS serum levels were significantly (LPS, IL-6, and TNF- α, *p* < 0.0001; IL-1β, *p*: 0.0012) reduced (T0 vs. T12) in Group A (Table 2), as well as lower levels of these markers being highlighted that in patients from Group A who reached im-IP compared with those who did not achieve this outcome (IL-1β, *p*: 0.0012; IL-6, *p*: 0.0034; TNF-α, *p*: 0.0274; LPS, *p*: 0.0178) (Figure 3B).

**Figure 4:**
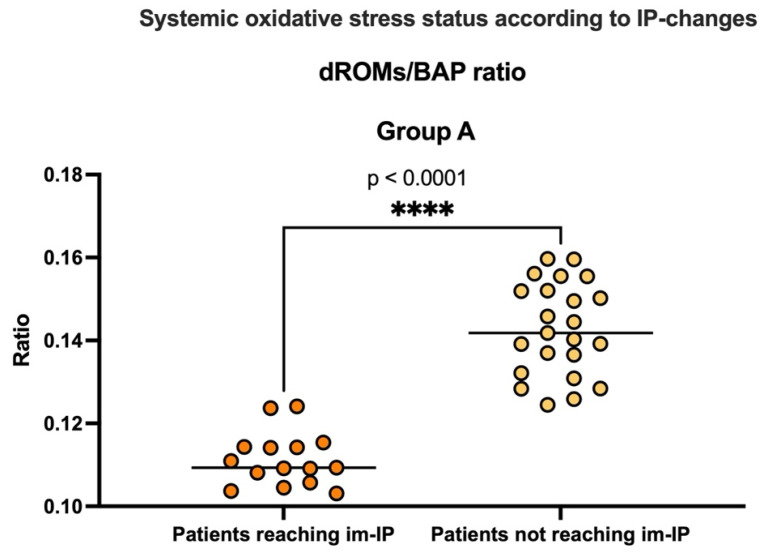
12-month systemic oxidative stress balance according to intestinal permeability (IP) changes. Systemic oxidative stress balance evaluated by dROM/BAP ratio at the end of the 12-month follow-up in Group A, according to the changes in intestinal permeability status. **** Mann–Whitney test analysis; im-IP: improved intestinal permeability; dROMs: derivatives of Reactive Oxygen Metabolites; BAP: Biological Antioxidant Potential.

Finally, unlike Group B, dROMs and BAP serum levels were significantly reduced (dROMs T0 vs. T12, *p* < 0.0001) and increased (BAP, T0 vs. T12, *p* < 0.0001) in Group A (Table 2). Relevantly, at the end of the 12-month follow-up, none of the Group B patients showed a compensated oxidative stress status (i.e., all individuals preserved a severe oxidative stress imbalance). Contrariwise, in Group A, none of the patients presented with a severe oxidative stress imbalance, and lower dROMs/BAP ratios were highlighted in patients reaching im-IP compared with those not achieving this outcome (dROMs/BAP ratio *p* < 0.0001) (Figure 4).

#### 3.3.2. Evaluation of Clinical Outcomes: Biochemical and Clinical Variable Modifications

Unlike Group B, a significant decrease in serum AST, ALT, and GGT levels was reported in Group A at the end of the study (Group A, AST, T0 vs. T12, *p*: 0.023; ALT, *p* < 0.0001; GGT, *p* < 0.0001) (Table 2). Moreover, focusing on metabolic dysfunction-related variables ahead of non-significant changes in BMI (Group A, T0 vs. T12, 29.82 ± 3.02 vs. 28.91 ± 2.98, *p*: 0.671; Group B: 29.49 ± 2.77 vs. 29.11 ± 2.78, *p*: 0.772), an improvement in hs-CRP, HDL, LDL, and insulin levels (all *p* < 0.0001) emerged in Group A (Table 2).

Consistently, unlike Group B, at the end of the study, a significant reduction (T0 vs. T12) of steatosis severity (CAP-T0 vs. T12, *p* < 0.0001) was revealed in Group A (Table 2), where an improvement of hepatic steatosis (i.e., reduction of at least 30% in CAP values, T0 vs. T12) was observed in 29/38 (76.31%) of patients (Group B, 6/35 individuals, 17.1%; chi-square, *p* < 0.0001) (Supplementary File S2 reports CAP continuous measures changes and S0-S3 categories changes in Group A) (Supplementary File S2).

**Figure 5:**
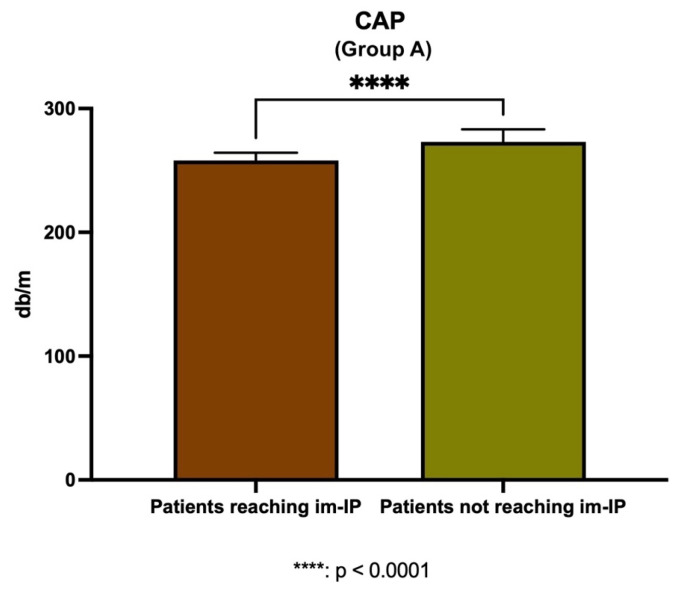
Variations in steatosis severity, according to the changes in intestinal permeability status (Group A). **** Mann–Whitney test analysis; CAP: controlled attenuation parameter; im-IP: improved intestinal permeability.

Interestingly, in Group A, patients reaching im-IP showed significantly lower CAP levels compared with individuals not achieving this outcome (*p* < 0.0001) (Figure 5).

Relevantly, multivariate logistic regression analysis (adjusted for sex, age, BMI, smoking, diabetes, MASLD-related drugs, and baseline CAP) revealed continuous water intake (aOR: 2.185, *p*: 0.001), im-IP achievement (aHR: 1.267, *p*: 0.021), and variations in systemic inflammation and oxidative stress status (IL-1β ∆_T0–T12_, aOR: 1.153, *p*: 0.030; IL-6 ∆_T0–T12_, aOR: 1.124, *p*: 0.039; TNF-α ∆_T0–T12_, aOR: 1.173, *p*: 0.004, LPS ∆_T0–T12_, aOR: 1.279, *p*: 0.002; dROMs/BAP ratio ∆_T0–T12_, aOR: 1.162, *p*: 0.005) as variables significantly associated with improvement in hepatic steatosis severity (Table 3) [sensitivity analyses summary in Supplementary Table S2 (Table S2)].

### 3.4. End of Water Wash-Out Period Evaluations

At the end of the water wash-out period, in Group A, no statistically significant differences emerged in the serum levels of metabolism-related biochemical variables, or in the fecal and serum levels of IP, SI, and oxidative stress markers (Table 4). Consistently and relevantly, all the patients who achieved im-IP after 12 months (at T12) preserved this status, and all these individuals continued to present without a severe oxidative stress imbalance.

Finally, in addition to no significant differences being observed in BMI (Group A, T12 vs. T18, 28.91 ± 2.98 vs. 28.42 ± 3.11, *p*: 0.682) or lifestyle habits (Table S1B), all the patients who obtained an improvement in hepatic steatosis severity at T12 preserved this status.

Table 4 reports all the 18-month follow-up evaluations (Group A), as well as the comparison between the assessed variables at the study time points (T12 and T18) (Table 4).

## 4. Discussion

In the era of continuously designing new drugs and emerging medications [36,37,38], lifestyle modification recommendations, including proper dietary habits, continue to represent a cornerstone in the management of MASLD, especially for the relevant proportion of patients without advanced-stage liver disease [17,30]. This feature appears even more relevant since foods have been demonstrated to influence several biological and metabolic functions [39], including inducing changes at the genetic and epigenetic level, just as, in turn, the individual genetic (and epigenetic) background can impact the ability to metabolize nutrients, ultimately contributing to the heterogeneity seen in patients with MASLD [16]. In this sense, the heterogeneity of clinical presentations of MASLD reflects the plethora of pathogenetic determinants promoting the onset and progression of liver disease, as well as the worsening of IR-related MD comorbidities in this setting [1,2,30]. In this scenario, recent evidence has underlined the crucial role of intestinal barrier dysfunction and microbial dysbiosis in the pathogenesis of MASLD [40], suggesting that interventions capable of restoring intestinal permeability integrity may mitigate SI, oxidative stress, and metabolic derangements [8,14,15]. In this context, non-pharmacological strategies aimed at enhancing gut barrier integrity are gaining interest, particularly given the lack of approved pharmacotherapies for early-stage MASLD [16,17]. In this context, dietary interventions have demonstrated potential in modulating disease activity, primarily through their impact on the gut microbiota, which serves as a pivotal interface between nutritional intake and immune regulation [16,18]. Such interventions contribute to the restoration of microbial eubiosis and reinforce the intestinal barrier’s integrity [18].

Fonte Essenziale^®^, a mineral water characterized by bicarbonate, sulfate, calcium, magnesium, and low sodium content, has previously demonstrated beneficial modulation of specific gut microbial taxa following six months of daily intake. Additionally, it has influenced key metabolic regulators, including gastrointestinal hormones (e.g., GLP-1, PYY) and hepatokines (e.g., fetuin-A, selenoprotein P), in individuals with hepatic steatosis [11].

Despite the well-established interplay between gut microbiota composition and intestinal barrier regulation [9,12], the potential of this water to influence IP, IP-associated SI, and oxidative stress remains uninvestigated. Notably, prior findings indicated that metabolic benefits vanished after a six-month washout, consistent with a causal effect mediated by barrier modulation, requiring the need for longer-term evaluation [11].

Considering this, the present study assessed the effects of long-lasting (12-month) daily Fonte Essenziale^®^ consumption, combined with specialist-guided lifestyle interventions (dietary and physical activity), on IP, IP-associated systemic inflammation, and oxidative stress in patients with MASLD, while concurrently exploring its impact on clinically relevant metabolic outcomes.

For this purpose, eighty-seven MASLD patients were enrolled and subsequently randomized into two groups: patients receiving the water (400 mL/day, in the morning on an empty stomach) and a controlled nutritional regimen for 12 months (Group A), followed by a 6-month water wash-out period and patients receiving only a controlled nutritional regimen for 12 months (Group B). The choice of a 12-month intervention was motivated by the need to explore not only short-term, reversible effects—as these were already shown in prior work [11]—but also the potential for sustained modulation of bowel permeability, SI, and metabolic outcomes in patients with MASLD. On the other side, the choice of this nutritional approach was guided by the following motivations: to minimize lifestyle habit-dependent intrapopulation study variability (that would have confounded gut dysbiosis/IP-related and inflammatory outcomes), aiming to find evidence of the “pure” effects of the water intake; and ethically, to offer to all the enrolled patients a valid approach, since Mediterranean-style diets and proper physical exercise have been consistently associated with improved metabolic control in MASLD patients [17,28,29,30]. Furthermore, the after-washout time point (T18) evaluation was limited to Group A to specifically assess the persistence of biological effects following cessation of the water intervention. As Group B did not receive mineral water supplementation, no post-intervention wash-out phase was considered, and therefore, no T18 assessment was planned for this arm.

The study was designed with a hierarchical structure of outcomes, reflecting both mechanistic and clinically relevant endpoints. By such a structuring of the outcomes, we linked mechanistic (IP, SI, oxidative stress) and clinical endpoints (steatosis and metabolism). This translational design allowed us to address both the pathophysiological underpinnings of the intervention and its potential impact on patient care.

The primary outcome was to investigate the variations in IP status in the two study groups by considering a well-validated panel of stool-assessable and circulating markers (fecal zonulin, serum occludin, serum claudin-1, and serum LPBp) in a dysmetabolic setting [13] as well as in the proportion of patients who achieved an improvement in IP after 12 months. Importantly, we operationalized im-IP as the concomitant normalization of two biomarkers [fecal zonulin (<110 ng/mL) and serum LBPp (<10 µg/mL)], based on previous evidence on this topic [13]. This dual definition was adopted because single-derived biological matrix markers may fluctuate due to technical or biological variability. In contrast, the simultaneous reduction in both was considered a more robust indicator of restored barrier integrity [13]. In line with previous evidence on the topic [6,13], while fecal zonulin and serum LBPp were used as primary quantitative markers to define altered and im-IP, occludin and claudin-1 were assessed as qualitative indicators of tight-junction integrity. Specifically, their expression levels were reported descriptively to support the overall interpretation of IP status. However, no predefined threshold values were applied to occludin and claudin-1 for categorical classification of IP alteration or improvement.

Interestingly, patients receiving mineral water showed a reduction in markers associated with increased permeability (fecal zonulin and serum LBPp), together with an increase in circulating tight-junction proteins such as occludin and claudin-1. Moreover, in addition to no baseline difference in IP status, in the group receiving water, a significant proportion of patients achieved an improvement in intestinal permeability after the intervention.

It is important to acknowledge the dual interpretative framework surrounding elevated serum levels of tight-junction proteins such as occludin and claudin-1. On one hand, increased circulating concentrations may reflect epithelial damage and tight junction disruption, as intracellular components are released into the bloodstream [13].

On the other hand, when measured using assays that selectively detect intact, functional protein isoforms—as in our study—elevated levels may instead indicate enhanced synthesis or stabilization of tight-junction structures, consistent with improved barrier integrity [13]. In our findings, the observed increases in serum occludin and claudin-1 occurred in parallel with significant reductions in fecal zonulin and serum LBPp, two validated markers of increased IP.

This concordance supports the interpretation that the rise in tight junction proteins reflects a regulatory, rather than degenerative, response—suggesting reinforcement of epithelial barrier function rather than its breakdown.

Altogether, these findings suggest a strengthening of the intestinal barrier, potentially mediated by the combined effects of bicarbonates and sulfates on the luminal microenvironment and epithelial homeostasis. In particular, the emerging results also appear in line with previous evidence supporting the contribution of bicarbonate and sulfate-ion-rich mineral waters in modulating luminal and intracellular pH, stimulating bile flow, and enhancing intestinal motility, ultimately preserving epithelial homeostasis [19,20]. Moreover, in the specific setting of MASLD, in murine models, calcium–sulfate–bicarbonate water intake has been associated with improved histopathological features and increased occludin expression in intestinal mucosa, suggesting a direct effect on tight-junction stabilization [21]. These mechanisms are consistent with the role of mineral ions in regulating tight-junction dynamics and epithelial permeability [22]. Therefore, focusing on the findings observed in the present research, changes in luminal pH and motility may have contributed to stabilizing tight junctions and reducing bacterial endotoxin translocation, in line with evidence that MASLD patients often show gut barrier dysfunction and dysbiosis [5,8,9].

In light of the abovementioned bacterial endotoxin systemic translocation, other relevant objectives of this research were represented by the investigations of changes in SI-related biomarkers (IL-1b, IL-6, TNF-α, LPS) and oxidative stress serum levels (dROMs, BAP, dROMs/BAP ratio) in the two study groups [26]. In patients receiving the water, after 12 months, a significant reduction in all pro-inflammatory markers was highlighted, as well as a decrease in the dROMs/BAP ratio, suggesting an improvement in both SI and oxidative balance status in comparison to the group of patients only receiving a controlled nutritional regimen. Moreover, within the water-receiving group, to further reinforce the association of these relevant findings with the obtained modifications in IP status, we evaluated SI and oxidative stress status according to changes in IP, revealing lower serum levels of all inflammatory markers and dROMs/BAP ratios in individuals who reached im-IP compared with those who did not achieve this outcome. The restoration of barrier function translated into a reduction in key pro-inflammatory mediators (LPS, IL-1, IL-6, and TNF-α). This is consistent with the “gut–liver axis” hypothesis, where increased permeability facilitates the entry of PAMPs into the portal circulation, activating Kupffer cells and ultimately sustaining chronic inflammation and oxidative stress [8,15]. By reducing this antigenic load, systemic cytokine activation was dampened, opening the way to improvements in hepatic and metabolic outcomes. These findings align with recent studies linking intestinal permeability and gut-derived inflammation to liver injury in MASLD/MASH [41].

Considering these obtained results, we further moved to investigate the secondary translational outcome of this research, evaluating the variations in hepatic steatosis severity in the two study groups along with changes in relevant biochemical metabolic variables. In this, a significant improvement in steatosis severity, simultaneously with a reduction in, among other metabolic variables, insulin and HDL serum levels, was highlighted in Group A. In this group, at baseline, no patients were in S0 (no steatosis), while at T12, 3 patients reached this category; after the intervention, the proportion of patients presenting in the S1 category (mild steatosis) at baseline increased from 15 to 19 patients, whereas the baseline S2 and S3 categories (moderate and severe steatosis) decreased at T12, suggesting an overall improvement of steatosis severity (Supplementary File S2). In this sense, the observed improvement in hepatic fat content (CAP), together with enhanced insulin sensitivity and a favorable modulation of the lipid profile, highlights the clinical relevance of IP and inflammation reduction. Notably, although exploratory, the reduction in ALT levels in the treatment group suggests an additional hepatocellular benefit, possibly linked to the attenuation of inflammatory stress.

On the other hand, despite the improvement in insulin levels, no significant changes in HOMA-IR were reported. This finding can be explained by the fact that HOMA-IR is derived from both fasting insulin and FPG, and the lack of a significant change in glycemia may have attenuated the overall variation in the index. Additionally, the distribution and variability of HOMA-IR values—often skewed and subject to inter-individual metabolic differences—may have limited the statistical power to detect subtle changes.

Altogether, the observed data support the idea that targeting the intestinal barrier may have downstream metabolic and hepatic benefits in MASLD, a theory also reinforced by recent experimental and clinical evidence [9,10]. In support of this, multivariate analysis, properly adjusted for confounding factors (including the administration of statins, GLP1-RA, and SGLT2-i), revealed water intake, im-IP, and the variations in systemic inflammation and oxidative stress status as variables significantly associated with improvement in hepatic steatosis.

### Strengths and Limitations of the Study

Our group previously demonstrated, in a short-term trial, that the same mineral water modulated gastrointestinal hormones and specific microbial taxa, with effects reversing after withdrawal [11].

In the present research, all the benefits were preserved after 6 months of water washout, suggesting that prolonging the intake is a valid cornerstone approach that is useful to avoid the vanishing of effects obtained after a short-lasting (i.e., 6 months) intervention, as adopted in the previous research [11]. The observed effects in Group A at T18 may reflect a combination of biological durability and non-specific influences. These may include sustained behavioral adherence, residual metabolic effects, or regression to the mean. Importantly, the absence of a parallel control assessment at T18 represents a relevant limit to definitively attributing the persistence of changes to the intervention alone.

Besides this novelty, the present randomized, longer-term study adds two new aspects: (i) it demonstrates that sustained consumption directly improves intestinal barrier function, systemic inflammation, and oxidative stress; (ii) it shows that these biological effects translate into measurable improvements in hepatic steatosis and the glucose–lipid metabolism. Thus, the present findings extend the evidence from endocrine/microbiota modulation to structural and functional barrier restoration with clinically relevant consequences. This is in line with previous research [11], which reported an increase in specific gut-microbiota species induced by mineral water intake, including, among others, *Subdoligranulum*, whose positive correlation with microbial richness and HDL-cholesterol levels, and negative correlation with fat mass, adipocyte diameter, IR, CRP, and IL-6 has been well demonstrated in humans [42]. In this sense, among the microbial taxa previously modulated by Fonte Essenziale^®^ intake, *Subdoligranulum* has emerged as a potentially beneficial genus associated with improved intestinal barrier function and reduced systemic inflammation. Prior observational studies have reported inverse correlations between *Subdoligranulum* abundance and markers of gut permeability and inflammation in autoimmune and dysmetabolic populations [43,44]. More recently, interventional data from dietary and probiotic research have suggested that targeted modulation of *Subdoligranulum* may contribute to barrier restoration and metabolic improvement in MASLD and related conditions [45]. Altogether, these findings support the hypothesis that mineral water-induced microbial shifts may play a mechanistic role in the observed improvements in IP and inflammatory status.

Another key element supporting the robustness of the observed findings is the good compliance observed throughout the intervention. Adherence to both the mineral water intake and the controlled diet was high, thanks to the simplicity of the regimen (a single morning administration) and the structured monitoring strategy adopted (periodic questionnaires, phone check-ins, and food diaries). This is clinically relevant, as in non-pharmacological trials, adherence often represents a major limitation to efficacy [2,38].

The good compliance achieved here suggests that prolonged intake of Fonte Essenziale^®^ is feasible in real-life settings and that the observed biological and clinical improvements may be realistically translated into daily practice.

This trial has limitations. First, no microbiota sequencing was performed; therefore, we cannot clarify whether the observed benefits were mediated by microbial changes, as previously reported. Similarly, short-chain fatty acids (SCFAs), key microbial metabolites involved in barrier integrity and metabolic regulation [5,41,46], were not assessed.

These analyses would have strengthened the mechanistic link between barrier restoration and clinical improvement by providing insight into the functional consequences of potential microbiota modulation.

Other limitations include the use of CAP rather than MRI-proton density and/or liver biopsy-proven fat quantification. While CAP is a validated non-invasive tool for assessing hepatic steatosis, it lacks the imaging/histological precision required to detect subtle changes in fat content and inflammation. Additionally, the exclusion of patients with AF may limit the generalizability of our findings. In this sense, while our findings are applicable to early-stage MASLD, the presence of portal hypertension and associated gut barrier alterations in AF may significantly modify the response to interventions targeting intestinal permeability [5,6]. In advanced disease stages, increased translocation of bacterial products and altered mucosal immunity may render barrier-targeted strategies less effective or mechanistically distinct [5,6]. Future studies should therefore evaluate the impact of mineral water intake and gut–liver axis modulation in AF populations, ideally incorporating stratification by fibrosis stage and portal pressure metrics to better delineate therapeutic responsiveness.

Finally, bowel permeability status was assessed using indirect biomarkers, such as serum zonulin, which, although validated and widely used in previous research, do not provide direct functional measurements of epithelial barrier integrity.

Nevertheless, these results open new perspectives. Future studies should integrate metagenomic and metabolomic analyses (including SCFAs), adopt more sensitive imaging techniques, and, given the systemic implications of IP modulation, explore other MASLD-related (including cardiovascular) clinical outcomes [5,41,46]. Moreover, subsequent trials could adopt a double-blind, placebo-controlled design to further strengthen causal inference. Finally, incorporating fecal permeability assays (e.g., lactulose/mannitol test), and advanced imaging modalities, such as MRI-proton density fat fraction (MRI-PDFF) for hepatic fat quantification, would provide a more comprehensive mechanistic and clinical assessment. These enhancements would allow for a more granular evaluation of water-mediated gut–liver axis modulation and its metabolic consequences in MASLD.

## 5. Conclusions

In the era of precision medicine, where multidisciplinary approaches to the management of chronic liver disorders are crucial, the daily and prolonged intake of a bicarbonate–sulfate–calcium–magnesium mineral water, Fonte Essenziale^®^, in combination with a controlled nutritional regimen, represents a valid strategy that significantly improves IP, low-grade SI, oxidative stress, and, in parallel, metabolic parameters and hepatic steatosis severity in patients with MASLD.

This intervention could represent a potentially useful non-pharmacological additional therapeutic weapon, in particular for those non-negligible portions of MASLD patients presenting with non-advanced stage liver disease who do not currently have access to emerging novel drug treatments [17].

Future studies incorporating microbiome sequencing, SCFA profiling, liver histology, and functional permeability assays would be instrumental in elucidating the mechanistic pathways underlying the observed effects and in confirming the therapeutic potential of Fonte Essenziale^®^ in MASLD management.

## Figures and Tables

**Table 1:** Baseline characteristics of study population groups.

Demographic, Anthropometric, and Clinical Data			
Variables	Group A (n. 44)	Group B (n. 43)	*p*-Value *
Sex (%, male)	23 (52.27%)	27 (62.79%)	n.s. **
Age (years) (mean ± SD)	56.05 ± 15.69	50.88 ± 18.82	n.s.
Smoke (%, yes)	19 (43.18%)	20 (46.51%)	n.s. **
Type 2 diabetes mellitus (%, yes)	21 (47.72%)	18 (41.86%)	n.s. **
Obesity (%, yes)	20 (45.45%)	18 (41.86%)	n.s.**
Body Mass Index (BMI) (mean ± SD)	29.82 ± 3.02	29.49 ± 2.77	n.s.
Essential arterial hypertension (%, yes)	29 (65.91%)	27 (62.79%)	n.s. **
Dyslipidemia (%, yes)	26 (59.09%)	24 (55.81%)	n.s. **
**Biochemical Data**			
**Variables (Mean ± SD)**	**Group A (n. 44)**	**Group B (n. 43)**	*p*-Value *
Aspartate aminotransferase (AST) (U/L) (n.v. 10–40)	53.20 ± 15.18	49.33 ± 9.21	n.s.
Alanine aminotransferase (ALT) (U/L) (n.v. 7–45)	59.81 ± 13.89	51.53 ± 17.05	n.s.
Gammaglutamil-transferase (GGT) (U/L) (n.v. 18–60)	77.64 ± 42.27	81.26 ± 64.69	n.s.
Alkaline Phosphatase (ALP) (U/L) (n.v. 44–145)	96.43 ± 15.52	89.19 ± 19.59	n.s.
Platelet (PLT) count (mm^3^) (n.v. 150–400)	221.1 ± 79.03	254.9 ± 38.38	n.s.
Bilirubin (mg/dL) (n.v. 0.3–1.2)	1.03 ± 0.07	0.89 ± 0.21	n.s.
Albumin (g/dL) (n.v. 3.5–5.0)	4.08 ± 0.38	4.21 ± 0.29	n.s.
High-sensitivity CRP (mg/L) (n.v. < 2.0)	2.11 ± 0.46	1.85 ± 0.65	n.s.
High-density lipoprotein (HDL) (mg/dL) (n.v. > 45)	40.48 ± 7.59	39.84 ± 10.43	n.s.
Low-density lipoprotein (LDL) (mg/dL) (n.v. < 120)	135.8 ± 33.31	144.2 ± 35.97	n.s.
Triglycerides (mg/dL) (n.v. < 150)	166.8 ± 37.48	179.5 ± 35.97	n.s.
Fasting Plasma Glucose (FPG) (mg/dL) (n.v. 70–99)	127.9 ± 14.86	129.4 ± 18.31	n.s.
Insulin (microu/L) (n.v. 2–11)	13.89 ± 3.41	11.74 ± 2.52	n.s.
HOMA-IR (n.v. < 2.5)	3.66 ± 1.83	3.79 ± 1.13	n.s.
**Non-Invasive Tools Assessing Liver Disease Progression Status**			
**Variables (Mean ± SD)**	**Group A (n. 44)**	**Group B (n. 43)**	*p*-Value *
Liver Stiffness Measurement (LSM) (kPa)	7.77 ± 1.39	7.16 ± 1.83	n.s.
Controlled Attenuation Parameter (CAP) (db/m)	278.1 ± 10.44	277.1 ± 10.31	n.s.
**Intestinal Permeability Markers**			
**Variables (Mean ± SD)**	**Group A (n. 44)**	**Group B (n. 43)**	*p*-Value *
Fecal zonulin (ng/mL) (n.v. < 110)	136.4 ± 45.71	126.4 ± 42.78	0.04
Serum occludin (ng/mL) (n.v. > 100)	248.5 ± 28.23	246.4 ± 32.12	n.s.
Serum claudin-1 (ng/mL) (n.v. > 1)	1.01 ± 0.27	1.09 ± 0.29	n.s.
Serum (LPBp) (microg/mL) (n.v. < 10)	12.11 ± 4.57	11.12 ± 3.47	n.s.
**Systemic Inflammation Assessment**			
**Variables**	**Group A (n. 44)**	**Group B (n. 43)**	*p*-Value *
Serum LPS (ng/mL) (n.v. < 0.1)	0.55 ± 0.27	0.52 ± 0.26	n.s.
Interleukin (IL)-1β (pg/mL) (n.v. < 3)	3.88 ± 1.12	3.82 ± 0.99	n.s.
Interleukin (IL)-6 (pg/mL) (n.v. < 10)	11.20 ± 0.97	10.58 ± 1.06	n.s.
Tumor Necrosis Factor-alpha (pg/mL) (n.v. < 8.1)	9.04 ± 0.60	9.03 ± 0.61	n.s.
**Systemic Oxidative Stress Assessment**			
**Variables**	**Group A (n. 44)**	**Group B (n. 43)**	*p*-Value *
dROMs (CARR-U) (n.v. < 300)	501.3 ± 109.4	513.2 ± 99.26	n.s.
BAP (mmol/L) (n.v. > 2200)	1714.1 ± 168.2	1684.2 ± 184.5	n.s.
dROMs/BAP ratio (n.v. < 0.1)	0.29 ± 0.08	0.30 ± 0.06	n.s.
Severe systemic oxidative stress imbalance (%)	39/44 (88.63%)	39/43 (90.69%)	n.s. **

Table 1 includes and compares the demographic, anthropometric, biochemical, clinical, intestinal permeability, systemic inflammation, and oxidative stress data of the two study groups. * Mann–Whitney test; ** Chi-square test; LDL: Low-density lipoprotein; HDL: High-density lipoprotein; CRP: C-reactive protein; HOMA-IR: Homeostatic Model Assessment of Insulin Resistance; LSM: Liver Stiffness Measurement; CAP: Controlled Attenuation Parameter; LPS: lipopolysaccharide; LPBp: LPS binding protein; CARR-U: Carratelli unit; dROMs: derivatives of Reactive Oxygen Metabolites; BAP: Biological Antioxidant Potential; SD: standard deviation; n.v.: normal values; n.s.: not statistically significant.

**Table 2:** Comparison of baseline and 12-month characteristics of study population groups.

Biochemical Data						
Variables (Mean ± SD)	Group A (n. 44) T0	Group A (n. 38) T12	*p*-Value *	Group B (n. 43) T0	Group B (n. 39) T12	*p*-Value *
AST (U/L) (n.v. 10–40)	53.20 ± 15.18	47.00 ± 4.49	**0.023**	49.33 ± 9.21	47.23 ± 3.36	n.s.
ALT (U/L) (n.v. 7–45)	59.81 ± 13.89	41.08 ± 4.41	**<0.0001**	51.53 ± 17.05	51.92 ± 3.93	n.s.
GGT (U/L) (n.v. 18–60)	77.64 ± 42.27	51.79 ± 4.45	**<0.0001**	81.26 ± 64.69	109.1 ± 16.77	n.s.
PLT count (mm^3^) (n.v. 150–400)	221.1 ± 79.03	244.2 ± 25.78	n.s.	254.9 ± 38.38	244.8 ± 2.95	n.s.
Bilirubin (mg/dL) (n.v. 0.3-1.2)	1.03 ± 0.07	1.18 ± 0.08	n.s.	0.89 ± 0.21	1.21 ± 0.07	n.s.
Albumin (g/dL) (n.v. 3.5–5.0)	4.08 ± 0.38	4.01 ± 0.06	n.s.	4.21 ± 0.29	3.99 ± 0.05	n.s.
Hs-CRP (mg/L) (n.v. < 2.0)	2.11 ± 0.46	1.51 ± 0.30	**<0.0001**	1.85 ± 0.65	1.81 ± 0.17	n.s.
HDL (mg/dL) (n.v. > 45)	40.48 ± 7.59	51.29 ± 2.25	**<0.0001**	39.84 ± 10.43	40.41 ± 2.99	n.s.
LDL (mg/dL) (n.v. < 120)	135.8 ± 33.31	99.68 ± 11.58	**<0.0001**	144.2 ± 35.97	125.7 ± 13.54	n.s.
Triglycerides (mg/dL) (n.v. < 150)	166.8 ± 37.48	161.6 ± 17.24	n.s.	179.5 ± 35.97	161.7 ± 14.41	n.s.
FPG (mg/dL) (n.v. 70–99)	127.9 ± 14.86	123.7 ± 7.59	n.s.	129.4 ± 18.31	122.9 ± 12.21	n.s.
Insulin (microu/L) (n.v. 2–11)	13.89 ± 3.41	9.21 ± 0.73	**<0.0001**	11.74 ± 2.52	12.38 ± 1.07	n.s.
HOMA-IR (n.v.< 2.5)	3.66 ± 1.83	2.81 ± 0.29	n.s.	3.79 ± 1.13	3.16 ± 0.28	n.s.
**Non-Invasive Tools Assessing Liver Disease Progression Status**						
**Variables (Mean ± SD)**	**Group A (n. 44)** **T0**	**Group A (n. 38)** **T12**	*p*-Value *	**Group B (n. 43)** **T0**	**Group B (n. 39)** **T12**	*p*-Value *
LSM (kPa)	7.77 ± 1.39	7.47 ± 0.60	n.s.	7.16 ± 1.83	7.45 ± 0.56	n.s.
CAP (db/m)	278.1 ± 10.44	264.8 ± 2.67	**<0.0001**	277.1 ± 10.31	279.4 ± 2.63	n.s.
**Intestinal Permeability Markers**						
**Variables (Mean ± SD)**	**Group A (n. 44)** **T0**	**Group A (n. 38)** **T12**	*p*-Value *	**Group B (n. 43)** **T0**	**Group B (n. 39)** **T12**	*p*-Value *
Fecal zonulin (ng/mL) (n.v. < 110)	136.4 ± 45.71	112.3 ± 12.01	**0.0163**	126.4 ± 42.78	135.2 ± 12.18	n.s.
Serum occluding (ng/mL) (n.v. > 100)	248.5 ± 28.23	290.1 ± 5.47	**<0.0001**	246.4 ± 32.12	249.3 ± 11.23	n.s.
Serum claudin-1 (ng/mL) (n.v. > 1)	1.01 ± 0.27	1.41 ± 0.05	**<0.0001**	1.09 ± 0.29	0.96 ± 0.17	n.s.
Serum (LPBp) (µg/mL) (n.v. < 10)	8.74 ± 1.81	7.42 ± 2.21	**<0.0001**	9.02 ± 1.75	12.08 ± 1.51	n.s.
**Systemic Inflammation Assessment**						
**Variables**	**Group A (n. 44)** **T0**	**Group A (n. 38)** **T12**	*p*-Value *	**Group B (n. 43)** **T0**	**Group B (n. 39)** **T12**	*p*-Value *
Serum LPS (ng/mL) (n.v < 0.1)	0.55 ± 0.27	0.19 ± 0.06	**<0.0001**	0.52 ± 0.26	0.59 ± 0.05	n.s.
IL-1β (pg/mL) (n.v. < 3)	3.88 ± 1.12	3.21 ± 0.18	**0.0012**	3.82 ± 0.99	3.80 ± 0.06	n.s.
IL-6 (pg/mL) (n.v. < 10)	11.20 ± 0.97	8.53 ± 0.28	**<0.0001**	10.58 ± 1.06	10.51 ± 0.27	n.s.
TNF-alpha (pg/mL) (n.v. < 8.1)	9.04 ± 0.60	7.44 ± 0.28	**<0.0001**	9.03 ± 0.61	9.92 ± 0.31	n.s.
**Systemic Oxidative Stress Assessment**						
**Variables**	**Group A (n. 44)** **T0**	**Group A (n. 38)** **T12**	*p*-Value *	**Group B (n. 43)** **T0**	**Group B (n. 39)** **T12**	*p*-Value *
dROMs (CARR-U) (n.v. < 300)	501.3 ± 109.4	264.9 ± 31.58	**<0.0001**	513.2 ± 99.26	553.1 ± 29.14	n.s.
BAP (mmol/L) (n.v. > 2200)	1714.1 ± 168.2	1898 ± 55.84	**<0.0001**	1684.2 ± 184.5	1604 ± 64.40	n.s.
dROMs/BAP ratio (n.v. < 0.1)	0.29 ± 0.08	0.13 ± 0.01	**<0.0001**	0.30 ± 0.06	0.34 ± 0.24	n.s.

Table 2 includes and compares the demographic, anthropometric, biochemical, clinical, intestinal permeability, systemic inflammation, and oxidative stress data of the two study groups at baseline and at the end of 12 months of intervention. * Wilcoxon test analysis; statistically significant differences are reported in bold. AST: aspartate aminotransferase; ALT: alanine aminotransferase; GGT: Gammaglutamil-transferase; ALP: alkaline phosphatase; Hs-CRP: high sensitivity C-reactive protein; LDL: Low-density lipoprotein; HDL: High-density lipoprotein; FPG: Fasting Plasma Glucose; HOMA-IR: Homeostatic Model Assessment of Insulin Resistance; LSM: Liver Stiffness Measurement; CAP: Controlled Attenuation Parameter; IL: interleukin; TNF: Tumor Necrosis Factor; LPS: lipopolysaccharide; LPBp: LPS binding protein; CARR-U: Carratelli unit; dROMs: derivatives of Reactive Oxygen Metabolites; BAP: Biological Antioxidant Potential; SD: standard deviation; n.v.: normal values; n.s.: not statistically significant.

**Table 3:** Predictors of hepatic steatosis improvement at T12.

Variable	Unadjusted OR [95% CI]	*p*-Value	Adjusted OR [95% CI]	*p*-Value
Age (years)	0.681 [0.48–0.96]	0.312	–	–
BMI (Kg/m^2^)	0.193 [0.11–0.22]	0.298	–	–
Baseline CAP (dB/m)	1.094 [0.95–1.31]	0.087	–	–
Type 2 Diabetes Mellitus	2.382 [2.21–2.79]	0.0015	n.s.	n.s.
Dyslipidemia	1.613 [1.45–1.79]	0.006	n.s.	n.s.
Physical exercise (h/day)	0.351 [0.26–0.68]	0.028	n.s.	n.s.
Water intake (compliance)	2.529 [2.35–2.91]	<0.0001	2.185 [2.01–2.34]	**0.001**
Improved IP	1.790 [1.31–2.04]	<0.0001	1.267 [1.14–1.89]	**0.021**
IL-1β Δ_T0–T12_	1.491 [1.23–1.66]	<0.0001	1.153 [1.09–1.27]	**0.030**
IL-6 Δ_T0–T12_	1.172 [1.10–1.42]	<0.0001	1.124 [1.07–1.23]	**0.039**
TNF-α Δ_T0–T12_	1.195 [1.11–1.54]	<0.0001	1.173 [1.09–1.33]	**0.004**
LPS Δ_T0–T12_	1.082 [1.02–1.39]	<0.0001	1.279 [1.20–1.41]	**0.002**
dROMs/BAP ratio Δ_T0–T12_	1.189 [1.13–1.61]	<0.0001	1.162 [1.11–1.32]	**0.005**

Logistic regression analysis for T12 improvement of hepatic steatosis severity (adjusted for sex, age, smoking, BMI, diabetes, MASLD-related drugs, and baseline CAP); statistically significant differences (*p* < 0.05) (in bold in the multivariate model). BMI: body mass index; IP: intestinal permeability; IL: interleukin; LPS: lipopolysaccharide; TNF: Tumor Necrosis factor; dROMs: derivatives of Reactive Oxygen Metabolites; BAP: Biological Antioxidant Potential; OR = Odds Ratio; CI = Confidence Interval; Δ_T0–T12_ indicated the variation T0 vs. T12; n.s.: not statistically significant.

**Table 4:** Comparison of 12-month characteristics and those after the wash-out period (Group A patients).

Biochemical Data			
Variables (Mean ± SD)	Group A (n. 38) T12	Group A (n. 35) T18	*p*-Value *
Aspartate aminotransferase (AST) (U/L) (n.v. 10–40)	47.00 ± 4.49	48.83 ± 1.21	n.s.
Alanine aminotransferase (ALT) (U/L) (n.v. 7–45)	41.08 ± 4.41	42.80 ± 1.28	n.s.
Gammaglutamil-transferase (GGT) (U/L) (n.v. 18–60)	51.79 ± 4.45	52.71 ± 0.95	n.s.
Alkaline Phosphatase (ALP) (U/L) (n.v. 44–145)	91.39 ± 4.78	92.71 ± 1.29	n.s.
Platelet (PLT) count (mm^3^) (n.v. 150–400)	244.2 ± 25.78	248.9 ± 6.23	n.s.
Bilirubin (mg/dL) (n.v. 0.3–1.2)	1.18 ± 0.08	1.13 ± 0.04	n.s.
Albumin (g/dL) (n.v. 3.5–5.0)	4.01 ± 0.06	4.15 ± 0.02	n.s.
High-sensitivity CRP (mg/L) (n.v. < 2.0)	1.51 ± 0.30	1.67 ± 0.08	n.s.
High-density lipoprotein (HDL) (mg/dL) (n.v. > 45)	51.29 ± 2.25	50.77 ± 1.62	n.s.
Low-density lipoprotein (LDL) (mg/dL) (n.v. < 120)	99.68 ± 11.58	93.66 ± 3.15	n.s.
Triglycerides (mg/dL) (n.v. < 150)	161.6 ± 17.24	167.5 ± 1.42	n.s.
Fasting Plasma Glucose (FPG) (mg/dL) (n.v. 70–99)	123.7 ± 7.59	126.5 ± 2.05	n.s.
Insulin (microu/l) (n.v. 2–11)	9.21 ± 0.73	10.24 ± 0.47	n.s.
HOMA-IR (n.v. < 2.5)	2.81 ± 0.29	3.01 ± 0.17	n.s.
**Non-Invasive Tools Assessing Liver Disease Progression Status**			
**Variables (Mean ± SD)**	**Group A (n. 38)** **T12**	**Group A (n. 35)** **T18**	*p*-Value *
Liver Stiffness Measurement (LSM) (kPa)	7.47 ± 0.60	7.41 ± 0.80	n.s.
Controlled Attenuation Parameter (CAP) (db/m)	264.8 ± 2.67	263.7 ± 1.66	n.s.
**Intestinal permeability markers**			
**Variables (Mean ± SD)**	**Group A (n. 38)** **T12**	**Group A (n. 35)** **T18**	*p*-Value *
Fecal zonulin (ng/mL) (n.v. 15–110)	112.3 ± 12.01	109.7 ± 5.72	n.s.
Serum occludin (ng/mL) (n.v. > 100)	290.1 ± 5.47	290.4 ± 3.03	n.s.
Serum claudin-1 (ng/mL) (n.v. > 1)	1.41 ± 0.05	1.472 ± 0.01	n.s.
Serum (LPBp) (microg/mL) (n.v. 0.5–10)	7.42 ± 2.21	8.42 ± 0.39	n.s.
**Systemic Inflammation Assessment**			
**Variables**	**Group A (n. 38)** **T12**	**Group A (n. 35)** **T18**	*p*-Value *
Serum LPS (ng/mL) (n.v. < 0.1)	0.19 ± 0.06	0.18 ± 0.02	n.s.
Interleukin (IL)-1β (pg/mL) (n.v. < 3)	3.21 ± 0.18	3.22 ± 0.07	n.s.
Interleukin (IL)-6 (pg/mL) (n.v. < 10)	8.53 ± 0.28	8.42 ± 0.16	n.s.
Tumor Necrosis Factor-alpha (pg/mL) (n.v. < 8.1)	7.44 ± 0.28	7.39 ± 0.12	n.s.
**Systemic Oxidative Stress Assessment**			
**Variables**	**Group A (n. 38)** **T12**	**Group A (n. 35)** **T18**	*p*-Value *
dROMs(CARR-U) (n.v. < 300)	264.9 ± 31.58	255.3 ± 17.41	n.s.
BAP (mmol/L) (n.v. > 2200)	1898 ± 55.84	1877 ± 27.32	n.s.
dROMs/BAP ratio (n.v. < 0.1)	0.13 ± 0.01	0.14 ± 0.02	n.s.

Table 4 includes and compares the demographic, anthropometric, biochemical, clinical, intestinal permeability, systemic inflammation, and oxidative stress data at the end of 12 months of intervention and at the end of the wash-out period in Group A. * Wilcoxon test analysis; LDL: Low-density lipoprotein; HDL: High-density lipoprotein; HOMA-IR: Homeostasis Model Assessment of Insulin Resistance; CRP: C-reactive protein; LSM: Liver Stiffness Measurement; CAP: Controlled Attenuation Parameter; LPS: lipopolysaccharide; LPBp: LPS binding protein; CARR-U: Carratelli unit; dROMs: derivatives of Reactive Oxygen Metabolites; BAP: Biological Antioxidant Potential; SD: standard deviation; n.v.: normal values; n.s.: not statistically significant.

## Data Availability

The data that support the findings of this study are available from the corresponding author upon reasonable request. The data are not publicly available due to ethical restrictions related to patient confidentiality and institutional data protection policies.

## Supplementary Materials

The following supporting information can be downloaded at: https://www.mdpi.com/article/10.3390/nu17213452/s1, Table S1A: Nutritional assessment in study population groups relative to the period of a 12-month controlled regimen; Table S1B: Nutritional assessment in Group A (relative to the interval 12-month–18-month controlled regimen). Table S2: Sensitivity analyses; File S1: International physical activity questionnaire; File S2: Changes in Controlled attenuation parameter (CAP) continuous values and CAP-categories after the intervention.

## Abbreviations

The following abbreviations are used in this manuscript:

ALT	Alanine Aminotransferase
AST	Aspartate Aminotransferase
BAP	Biological Antioxidant Potential
BMI	Body Mass Index
BP	Blood Pressure
CRP	C-Reactive Protein
d-ROMs	Reactive Oxygen Metabolites Test
γGT	Gamma-Glutamyl Transferase
GLP-1	Glucagon-Like Peptide-1
HDL	High-Density Lipoprotein Cholesterol
HOMA-IR	Homeostasis Model Assessment of Insulin Resistance
im-IP	Impaired Intestinal Permeability
in-IP	Intact Intestinal Permeability
IP	Intestinal Permeability
LDL	Low-Density Lipoprotein Cholesterol
MASLD	Metabolic Dysfunction-Associated Steatotic Liver Disease
MD	Metabolic Dysfunction
PLT	platelet
PYY	Peptide Tyrosine–Tyrosine
RCF	Relative Centrifugal Force
SI	Systemic Inflammation
SOS	Systemic Oxidative Stress
TG	Triglycerides
U-CARR	Carratelli Units (unit of d-ROMs test)
