# Supplementary material for: Beneficial Effects of Long-Lasting Bicarbonate–Sulfate–Calcium–Magnesium Water Intake on Metabolic Dysfunction-Associated Steatotic Liver Disease (MASLD)-Related Outcomes via Impacting Intestinal Permeability (IP), IP-Related Systemic Inflammation, and Oxidative Stress
Source: Nutrients. 2025 Oct 31;17(21):3452. doi: 10.3390/nu17213452 (PMC12609797; doi:10.3390/nu17213452)
Supplement: Supplementary file 1 [file nutrients-17-03452-s001.zip › Supplementary/Etich approval IRB + blank informed consent/ENG Blank informed consent-2 copia.pdf]

INFORMED CONSENT FORM FOR THE STUDY

**Operational Unit Manager**	
**Area References**	

The undersigned ______________________________

Born on ______________ to ______________________ resident in ______________________

Participant Declaration:

I have received detailed and understandable explanations regarding the proposed study. I have read and understood all the information contained in the information leaflet. I have had the opportunity to speak with the doctor and ask all the necessary questions. I am satisfied with the answers I received. I have had sufficient time to reflect on the invitation and make a decision. I understand that I can withdraw my consent at any time (verbally or in writing) without providing any reason and without this impacting my usual healthcare.

I have received a copy of the study information, the informed consent form, the privacy policy, and the consent to the processing of personal data. The originals are kept at the center.
I received the information letter from the practice to forward to my doctor.

I declare that I am voluntarily willing to participate in the above-mentioned study, therefore:

I **consent to the processing of my demographic, clinical, anthropometric and therapeutic data**

__________ ______________________________
Date Patient's signature

__________ ______________________________
Date Signature of the doctor who informed the patient

__________ ______________________________
Date Signature of the participant's legal representative/guardian
(only in case of patient inability)
