# Supplementary material for: Beneficial Effects of Long-Lasting Bicarbonate–Sulfate–Calcium–Magnesium Water Intake on Metabolic Dysfunction-Associated Steatotic Liver Disease (MASLD)-Related Outcomes via Impacting Intestinal Permeability (IP), IP-Related Systemic Inflammation, and Oxidative Stress
Source: Nutrients. 2025 Oct 31;17(21):3452. doi: 10.3390/nu17213452 (PMC12609797; doi:10.3390/nu17213452)
Supplement: Supplementary file 1 [file nutrients-17-03452-s001.zip › Supplementary/Etich approval IRB + blank informed consent/Ethics approval.pdf]

AOU Università della Campania Vanvitelli - Prot. 0001933/i del 20/01/2023 10.03 Registrato da: Comitato Etico
20/01/2023 10.03-20230001933

AOV: Azienda Ospedaliera Universitaria Luigi Vanvitelli

OSPEDALI DEI COLLI
MONALDI - COTUGNO - C.T.O.
"AZIENDA OSPEDALIERA SPECIALISTICA DEI COLLI"
Monaldi - Cotugno - C.T.O.

Comitato Etico Università degli Studi della Campania "Luigi Vanvitelli" - Azienda Ospedaliera Universitaria "Luigi Vanvitelli" - AORN "Ospedali dei Colli"

Al Responsabile dello studio
Prof. Alessandro Federico

Al Direttore Sanitario AOU "Luigi Vanvitelli"
Dott. Pasquale Di Girolamo Faraone

Oggetto: parere dello studio dal titolo: "Effetto dell'acqua fonte essenziale® sull'alterata permeabilità intestinale come denominatore comune nella patogenesi della sindrome dell'intestino irritabile (IBS) e della malattia del fegato grasso associata a disfunzione metabolica (MAFLD)" Versione 2.0 del 27/12/2022; Responsabile dello Studio: Prof. Alessandro Federico afferente al DAI di Chirurgia, Ortopedia e Epatogastroenterologia.

Nella seduta del 10.01.2023 il Comitato Etico Università degli Studi della Campania "Luigi Vanvitelli" - Azienda Ospedaliera Universitaria "Luigi Vanvitelli"- AORN "Ospedali dei Colli" che opera da un punto di vista organizzativo ed operativo in conformità alla GCP, alle leggi e alle disposizioni normative applicabili e così composto ai sensi del DM 08.02.2013:

Prof. Alfano Roberto	Esperto Clinico del settore biomedico	PRESENTE
Dott. D'Alto Michele	Clinico	PRESENTE
Prof. D'Amico Michele	Farmacologo	PRESENTE
Dott. D'Aniello Carmine	Clinico	PRESENTE
Dott. De Luca Alfonso	Rappresentante professioni sanitarie	ASSENTE
Dott. ssa De Rimini Maria Luisa	Esperto di nuove procedure diagnostiche terapeutiche invasive e seminvasive	PRESENTE
Dott. Di Girolamo Faraone Pasquale	Direttore Sanitario "AOU Luigi Vanvitelli"	ASSENTE
Dott. Fiorentino Giuseppe	Direttore Sanitario "AORN Ospedali dei Colli"	ASSENTE
Dott.ssa Galdo Maria	Farmacista SSR	PRESENTE
Dott.ssa Giordano Maria Luisa	Farmacista SSR	PRESENTE

Dott.ssa Iommelli Chiara	Esperta di studi clinici	PRESENTE
Dott. Landolfi Roberto	Esperto in Bioetica	PRESENTE
Dott. Lima Guglielmo	Esperto in materie giuridiche ed assicurative	PRESENTE
Prof. Marfella Raffaele	Clinico, esperto anche in genetica	PRESENTE
Dott.ssa Marrone Francesca	Farmacista SSR	PRESENTE
Dott. ssa Messina Antonietta	Esperto in nutrizione	PRESENTE
Dott. Montesarchio Vincenzo	Clinico	PRESENTE
Prof.ssa Morgillo Floriana	Clinico	ASSENTE
Prof. Napoli Claudio	Clinico	PRESENTE
Prof.ssa Pace Maria Caterina	Esperto di nuove procedure diagnostiche terapeutiche invasive e seminvasive	PRESENTE
Dott.ssa Papa Nunzia	Farmacista SSR Esperto in dispositivi medici	PRESENTE
Dott. Parrella Roberto	Clinico	ASSENTE
Prof. Patroni Griffi Andrea	Esperto in materie giuridiche ed assicurative	PRESENTE
Prof. Perrotta Silverio	Pediatra, esperto anche in genetica	PRESENTE
Dott.ssa Piegari Ester	Medico Legale	ASSENTE
Dott. Schiavo Vincenzo	MMG	PRESENTE
Prof.ssa Signoriello Simona	Biostatistico	ASSENTE
Prof. Tessitore Alessandro	Clinico	ASSENTE
Prof. Tolone Salvatore	Clinico	PRESENTE
Prof. Vitale Antonio	Pediatra	PRESENTE
Dott. Zofra Sergio	Rappresentante del volontariato/associazioni	PRESENTE

Esaminata la documentazione presentata dal Prof. Alessandro Federico, assunta al protocollo dell'A.O.U. Luigi Vanvitelli n. 38980/2022 dello studio dal titolo: "Effetto dell'acqua fonte essenziale® sull'alterata permeabilità intestinale come denominatore comune nella patogenesi della sindrome dell'intestino irritabile (IBS) e della malattia del fegato grasso associata a disfunzione

metabolica (MAFLD)" Versione 2.0 del 27/12/2022; Responsabile dello Studio: Prof. Alessandro Federico afferente al DAI di Chirurgia, Ortopedia e Epatogastroenterologia:

1. lettera di trasmissione del versione 2.0 del 27/12/2022;
2. griglia riassuntiva dello studio del 02/11/2022;
3. CV dello sperimentatore principale del 31/10/2022;
4. CV dott.ssa Gravina del 22/02/2019;
5. CV dott. Marcello Dallio del 10/10/22;
6. dichiarazione dello sperimentatore di disporre del tempo e dei mezzi e dichiarazione di assenza di conflitto di interesse personale;
7. dichiarazione dello sperimentatore di attenersi alla dichiarazione di Helsinki Versione 1.0 del 02/11/2022;
8. delibera del DAI del 7.11.2022;
9. protocollo di studio versione 2.0 del 27/12/2022;
10. sinossi del protocollo versione 2.0 del 27/12/2022;
11. informativa per il trattamento dei dati per scopi di sperimentazione clinica per gli studi no profit versione 1.0 del 02/11/2022;
12. foglio informativo per paziente versione 2.0 del 27/12/2022;
13. modulo di consenso informato allo studio (versione 1, del 02/11/2022);
14. lettera informativa per il medico di medicina generale versione 1, del 02/11/2022;
15. modulo per il consenso al trattamento dei dati personali;
16. polizza assicurativa 28/06/2022;
17. scheda clinica per la raccolta dati CRF;
18. brochure fonte essenziale;
19. manuale d'uso fonte essenziale;
20. bozza di convenzione economica del 26/10/2022;
21. elenco del Referente e coordinatore dello studio e dei partecipanti allo studio 02/11/2022.
22. dettagli sulle modalità di randomizzazione e sulla conduzione dello studio in cieco;
23. lettera di risposte di richieste alle modifiche del 27/12/2022;
24. check list di validazione.

Accertata la sussistenza del numero legale e del numero degli aventi diritto al voto;

Considerato che i componenti del Comitato Etico dichiarano di astenersi dal pronunciarsi su quegli studi per i quali possa sussistere un conflitto di interesse di tipo diretto o indiretto;

Verificati i seguenti aspetti:

- Conformità ai principi di Buona Pratica Clinica (GCP);
- Rispetto delle regole di riservatezza e confidenzialità previste delle disposizioni normative applicabili;
- Salvaguardia dei diritti di sicurezza e benessere dei soggetti partecipanti allo studio;
- Validità scientifica e giustificazione etica dello studio;
- Correttezza del disegno dello studio e delle procedure di reclutamento dei soggetti;
- Completezza e chiarezza del modulo di consenso informato;
- Appropriatezza delle indagini e/o terapie proposte;
- Adeguatezza del rapporto rischi prevedibili/benefici attesi;
- Adeguatezza degli spazi e delle strutture e tecnologie disponibili;
- Idonea qualificazione del Responsabile dello studio.

Visto il regolamento del Comitato Etico approvato nella seduta del giorno 31.03.2022;

Letta la valutazione del relatore designato per l'approfondimento dello studio;

ESPRIME PARERE FAVOREVOLE

all'effettuazione dello studio di cui sopra, che dovrà essere condotto ai sensi della normativa vigente in materia e si raccomanda che venga salvaguardata l'autonomia del promotore indispensabile stante la natura no profit dello studio.

Si ricorda che è obbligo notificare al Comitato Etico:

- data di arruolamento del primo paziente;
- stato di avanzamento dello studio, con cadenza annuale, corredato da una relazione scritta;
- fine del periodo di arruolamento;
- data di conclusione dello studio a livello locale ed a livello globale;
- risultati dello studio, entro un anno dalla conclusione dello stesso.

IL PRESIDENTE DEL COMITATO ETICO
Prof. Claudio Napoli

COMITATO ETICO
Università degli Studi della Campania "Luigi Vanvitelli"
Azienda Ospedaliera Universitaria "Luigi Vanvitelli"
A.O.R.N. "Ospedali dei Colli"
