# Supplementary material for: Beneficial Effects of Long-Lasting Bicarbonate–Sulfate–Calcium–Magnesium Water Intake on Metabolic Dysfunction-Associated Steatotic Liver Disease (MASLD)-Related Outcomes via Impacting Intestinal Permeability (IP), IP-Related Systemic Inflammation, and Oxidative Stress
Source: Nutrients. 2025 Oct 31;17(21):3452. doi: 10.3390/nu17213452 (PMC12609797; doi:10.3390/nu17213452)
Supplement: Supplementary file 1 [file nutrients-17-03452-s001.zip › Supplementary/Etich approval IRB + blank informed consent/ITA Blank informed consent-2.pdf]

Azienda Ospedaliera Universitaria
Università degli Studi della Campania "L. Vanvitelli"
DAI di CHIRURGIA, ORTOPEDIA ED EPATOGASTROENTEROLOGIA

AOV: Azienda Ospedaliera Universitaria *Luigi Vanvitelli*

UNITA' OPERATIVA COMPLESSA DI EPATO-GASTROENTEROLOGIA

MODULO DI CONSENSO INFORMATO ALLO STUDIO

**Responsabile Unità Operativa**	
**Riferimenti di Area**	

Il sottoscritto ____

Nato/a il _________________ a ________________________ residente a ___________________________

Dichiarazione del Partecipante:

Ho ricevuto spiegazioni dettagliate e comprensibili in merito allo studio proposto. Ho letto e compreso tutti i contenuti dell'informativa. Ho avuto l'opportunità di parlare con il medico e di porre tutte le domande necessarie. Sono soddisfatto delle risposte ricevute. Ho avuto abbastanza tempo per riflettere sull'invito e prendere una decisione. Sono consapevole del fatto che posso ritirare il mio consenso in qualsiasi momento (verbalmente o per iscritto) senza dare alcuna giustificazione e senza che ciò abbia un impatto sulla mia abituale assistenza sanitaria.

Ho ricevuto una copia dell'informativa dello studio, del modulo di consenso informato, dell'informativa e del consenso al trattamento dei dati personali. Gli originali sono conservato presso il centro.
Ho ricevuto la lettera informativa allo studio da trasmettere al medico curante.

Dichiaro di essere volontariamente disposto a partecipare allo studio sopra menzionato, pertanto:

☐ **Acconsento al trattamento dei miei dati demografici, clinici, antropometrici e terapeutici**

________________ ____
Data Firma del paziente

________________ ____
Data Firma del medico che ha informato il paziente

________________ ____
Data Firma del rappresentante legale/tutore del partecipante
(solo in caso di impossibilità del paziente)
