# Supplementary material for: Beneficial Effects of Long-Lasting Bicarbonate–Sulfate–Calcium–Magnesium Water Intake on Metabolic Dysfunction-Associated Steatotic Liver Disease (MASLD)-Related Outcomes via Impacting Intestinal Permeability (IP), IP-Related Systemic Inflammation, and Oxidative Stress
Source: Nutrients. 2025 Oct 31;17(21):3452. doi: 10.3390/nu17213452 (PMC12609797; doi:10.3390/nu17213452)

Differences of CAP values: baseline (T0) vs after the intervention (T12) – Group A		
**Variables (mean ± Standard Deviation)**	**Group A (n.44) T0**	**Group A (n.38) T12**
CAP (db/m)	278.1 ± 10.44	264.8 ± 2.67

Controlled Attenuation Parameter (CAP) categories distribution (Group A)		
**CAP category**	**T0 (n = 44)**	**T12 (n = 38)**
**S0**	0	3
**S1**	15	19
**S2**	11	9
**S3**	18	7
	**T12 mild (S0–S1)**	**T12 moderate/severe (S2–S3)**
T0 lieve (S0-S1)	15	10
T0 moderata/severa (S2-S3)	1	18

McNemar test: $p = 0.011$

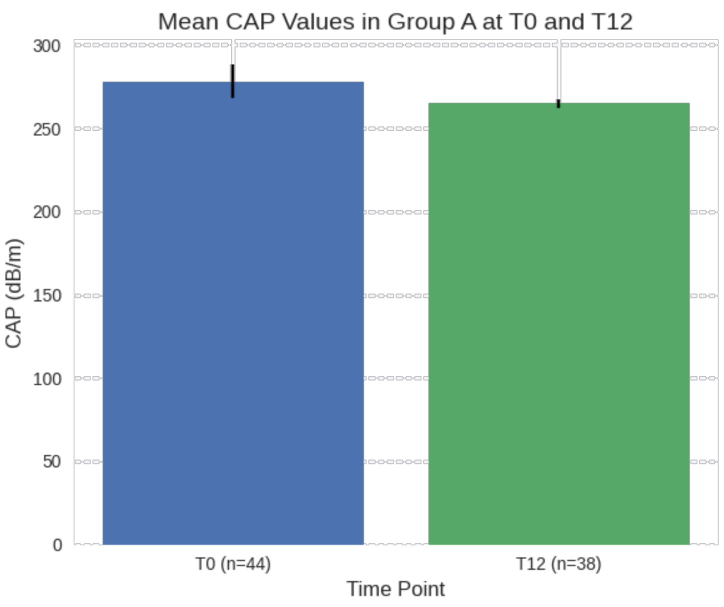

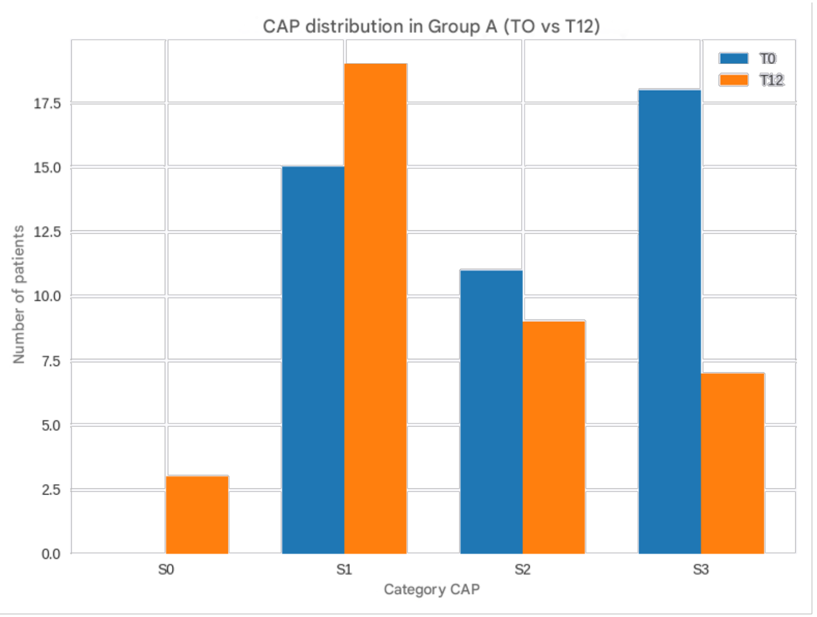

Supplement: Supplementary file 1 [file nutrients-17-03452-s001.zip › Supplementary/Supplementary File S2.pdf]
