# Supplementary material for: Beneficial Effects of Long-Lasting Bicarbonate–Sulfate–Calcium–Magnesium Water Intake on Metabolic Dysfunction-Associated Steatotic Liver Disease (MASLD)-Related Outcomes via Impacting Intestinal Permeability (IP), IP-Related Systemic Inflammation, and Oxidative Stress
Source: Nutrients. 2025 Oct 31;17(21):3452. doi: 10.3390/nu17213452 (PMC12609797; doi:10.3390/nu17213452)
Supplement: Supplementary file 1 [file nutrients-17-03452-s001.zip › Supplementary/Table S1 Supplementary Table 1.pdf]

Supplementary Table 1A. Nutritional assessment in study population groups relative to the period of a 12-month controlled regimen

**Variables**	**Group A**	**Group B**	***p*-value of the comparison between the study groups**
Physical activity (hours/week in the 12 months) (mean ± SD) (interval T0-T12)	6.2 ± 1.8	6.2 ± 1.1	Group A vs Group B: n.s.
Patients (%) on "active physical exercise" (interval T0-T12)	74.8	72.4	Group A vs Group B: n.s.
Daily intake (Kilocalories / day) (mean ± SD) (interval T0-T12)	2304 ± 815.3	2302 ± 409.2	Group A vs Group B: n.s.
Carbohydrates (Kilocalories) (mean ± SD) (interval T0-T12)	1071± 233.5	1074 ± 224.4	Group A vs Group B: n.s.
Lipids (Kilocalories) (mean ± SD) (interval T0-T12)	576.4 ± 241.5	581.7 ± 132.6	Group A vs Group B: n.s.
Proteins (Kilocalories) (mean ± SD) (interval T0-T12)	656.3 ± 201.5	654.6 ± 198.5	Group A vs Group B: n.s.

MASLD: Metabolic Dysregulated-Associated Steatotic Liver Disease; SD: standard deviation. In the case of non-normal or normal distribution, the Mann-Whitney test or t-test was performed to compare the continuous variables among different study groups. Statistically significant differences (p<0.05) are reported in bold.

Supplementary Table 1B. Nutritional assessment in Group A (relative to the interval 12-month – 18-month controlled regimen)

**Variables**	**Group A (T0-T12)**	**Group A (T12-T18)**	***p*-value of the comparison between the study groups**
Physical activity (hours/week in the 12 months) (mean ± SD) (interval T0-T12)	6.2 ± 1.8	6.1 ± 1.2	Group A T12 vs Group A T18: n.s.
Patients (%) on "active physical exercise" (interval T0-T12)	74.8	75.6	Group A T12 vs Group A T18: n.s.
Daily intake (Kilocalories / day) (mean ± SD) (interval T0-T12)	2304 ± 815.3	2311 ± 411.5	Group A T12 vs Group A T18: n.s.
Carbohydrates (Kilocalories) (mean ± SD) (interval T0-T12)	1071± 233.5	1075 ± 226.8	Group A T12 vs Group A T18: n.s.
Lipids (Kilocalories) (mean ± SD) (interval T0-T12)	576.4 ± 241.5	584.9 ± 135.4	Group A T12 vs Group A T18: n.s.
Proteins (Kilocalories) (mean ± SD) (interval T0-T12)	656.3 ± 201.5	651.8 ± 177.5	Group A T12 vs Group A T18: n.s.

MASLD: Metabolic Dysregulated-Associated Steatotic Liver Disease; SD: standard deviation. In the case of non-normal or normal distribution, the Mann-Whitney test or t-test was performed to compare the continuous variables among different study groups. Statistically significant differences (p<0.05) are reported in bold.
