# Supplementary material for: Beneficial Effects of Long-Lasting Bicarbonate–Sulfate–Calcium–Magnesium Water Intake on Metabolic Dysfunction-Associated Steatotic Liver Disease (MASLD)-Related Outcomes via Impacting Intestinal Permeability (IP), IP-Related Systemic Inflammation, and Oxidative Stress
Source: Nutrients. 2025 Oct 31;17(21):3452. doi: 10.3390/nu17213452 (PMC12609797; doi:10.3390/nu17213452)
Supplement: Supplementary file 1 [file nutrients-17-03452-s001.zip › Supplementary/Table S2 Supplementary Table 2.pdf]

Multinomial logistic regression analysis showing the variables significantly associated with hepatic steatosis improvement.

	Physical exercise	Water intake	Improved IP	IL-1β ΔT0–T12	IL-6 ΔT0–T12	TNF-α ΔT0–T12	LPS ΔT0–T12	dROMs/BAP ratio ΔT0–T12
Age								
< median age	0.76 (0.65 - 0.94)	0.51 (0.43 - 0.82)	1.48 (0.27 - 0.87)	1.47 (0.28 - 0.79)	1.66 (0.50 - 0.86)	1.68 (1.21 - 2.33)	1.69 (1.23 - 2.32)	1.84 (0.71 - 0.99)
≥ median age	0.63 (0.40 - 0.98)	0.52 (0.26 - 1.04)	1.33 (0.10 - 1.08)	0.18 (0.03 - 1.15)	0.52 (0.28 - 0.99)	1.33 (0.98 - 1.79)	1.91 (0.96 - 3.81)	1.77 (0.55 - 1.08)
Sex								
Female	0.74 (0.64 - 0.91)	0.75 (0.65 - 0.92)	1.60 (0.35 - 1.03)	1.54 (0.36 - 0.80)	1.73 (0.57 - 0.95)	1.16 (1.02 - 1.33)	1.29 (1.08 – 1.52)	1.89 (0.77 - 1.02)
Male	0.81 (0.64 - 1.02)	0.84 (0.69 - 1.09)	1.38 (0.18 - 0.84)	1.27 (0.079 - 0.92)	1.47 (0.27 - 0.82)	2.11 (0.79 – 11.39)	2.24 (0.87 – 12.09)	0.67 (0.42 - 1.06)
BMI								
< median BMI	0.72 (0.58 - 0.93)	0.61 (0.48 - 0.85)	1.53 (0.27 - 1.05)	1.35 (0.18 - 0.70)	1.67 (0.46 - 0.97)	1.56 (1.18 - 2.06)	1.57 (1.18 - 2.09)	1.86 (0.74 - 1.00)
≥ median BMI	0.80 (0.66 - 0.97)	0.55 (0.33 - 0.90)	1.56 (0.32 - 0.98)	0.56 (0.35 - 0.87)	1.79 (0.49 - 0.98)	1.80 (1.03 - 3.14)	1.83 (1.11 - 3.01)	0.88 (0.70 - 1.09)
Type 2 diabetes								
No	0.83 (0.68 - 0.97)	0.57 (0.37 - 0.85)	1.58 (0.34 - 0.99)	1.46 (0.28 - 0.76)	1.59 (0.41 - 0.84)	1.76 (1.18 - 2.64)	1.79 (1.17 - 2.73)	1.85 (0.70 - 1.04)
Yes	0.59 (0.37 - 0.94)	0.68 (0.50 - 0.91)	1.40 (0.18 - 0.92)	1.37 (0.16 - 0.85)	1.65 (0.44 - 0.97)	1.29 (1.05 - 1.60)	1.48 (1.10 - 1.99)	1.78 (0.61 - 0.99)
Smoking								
No	0.76 (0.65 - 0.91)	0.62 (0.45 - 0.83)	1.58 (0.36 - 0.95)	1.50 (0.33 - 0.75)	1.68 (0.52 - 0.89)	1.34 (1.12 - 1.61)	1.63 (1.21 - 2.21)	1.84 (0.73 - 0.97)
Yes	0.83 (0.67 - 1.03)	0.69 (0.50 - 0.96)	1.15 (0.023 - 1.02)	1.34 (0.13 - 0.90)	1.51 (0.29 - 0.90)	1.50 (0.99 - 2.29)	1.45 (1.04 - 2.01)	1.88 (0.70 - 1.11)
CAP								
< median LSM	0.74 (0.63 - 0.92)	0.61 (0.51 - 0.82)	1.43 (0.24 - 0.78)	1.30 (0.14 - 0.64)	1.59 (0.42 - 0.82)	1.53 (1.20 - 1.96)	1.55 (1.22 - 1.98)	1.69 (0.46 - 1.04)
≥ median LSM	0.84 (0.69 - 1.02)	0.74 (0.55 - 0.99)	1.52 (0.21 - 1.31)	1.65 (0.44 - 0.97)	1.73 (0.50 - 1.05)	1.17 (0.96 - 1.42)	1.36 (1.01 - 1.82)	1.85 (0.64 - 1.14)

Model adjusted for age, sex, smoking, BMI, type 2 diabetes, CAP, MASLD-related drugs (see next page*)

*Sensitivity analysis summary [relatively to multivariate logistic regression for T12 hepatic steatosis improvement, adjusted for MASLD-related medications (Group A)]

Medication	T2-Improved (%)	T12-Not Improved (%)	p-value (Chi-square)
Statins (n=15)	62.5%	37.5%	0.412
No Statins (n= 29)	64.8%	35.2%	
GLP-1RA (n=9)	66.7%	33.3%	0.298
No GLP-1RA (n=35)	63.1%	36.9%	
SGLT2-i (n=11)	65.2%	34.8%	0.337
No SGLT2-i (n=33)	63.9%	36.1%	

Multi-adjusted Odds ratios (OR) with 95% CI of Steatosis Improvement (at T12).

Variable	Adjusted OR [95% CI]	p-value
IL-1β ΔT0–T12	1.13 [1.07–1.26]	0.033
IL-6 ΔT0–T12	1.11 [1.06–1.22]	0.037
TNF-α ΔT0–T12	1.17 [1.10–1.33]	0.004
LPS ΔT0–T12	1.28 [1.21–1.43]	0.002
dROMs/BAP ratio ΔT0–T12	1.16 [1.11–1.32]	0.005
Statin use	1.02 [0.89–1.18]	0.743
GLP-1RA use	1.09 [0.94–1.27]	0.211
SGLT2-i use	1.07 [0.93–1.23]	0.264
Sex (male)	0.93 [0.80–1.08]	0.346
Age (years)	0.96 [0.90–1.02]	0.278
BMI (Kg/m²)	0.91 [0.84–1.00]	0.071
Type 2 Diabetes Mellitus	1.06 [0.93–1.23]	0.309
Baseline CAP (dB/m)	1.07 [0.96–1.20]	0.226

Model adjusted for sex, age, BMI, diabetes, baseline CAP, and listed medications. OR = Odds Ratio; CI = Confidence Interval. BMI: Body mass index; IL: Interleukin; TNF: Tumor necrosis factor; GLP1-RA: glucagon-like peptide-1 receptor agonist; SGLT2-i: Sodium-Glucose Transport 2 inhibitors.
